# Assessing the effectiveness of demand-management-technology in reducing CO_2_ from urban passenger transportation

**DOI:** 10.1186/s13021-025-00343-y

**Published:** 2025-11-10

**Authors:** Xin Li, Yongsheng Qian, Jianxin Wang, Minan Yang, Junwei Zeng, Xiaofang Xie

**Affiliations:** 1 School of Traffic and Transportation, Lanzhou Jiaotong University, No. 88, Anning West RoadGansu Province, LanzhouAnning, 730070 China; 2 School of Architecture and Urban Planning, Lanzhou Jiaotong University, Lanzhou, 730070 China; 3 School of Tourism and History and Culture, YiLi Normal University, Xinjiang, Yining, 835000 China

**Keywords:** Urban passenger transportation, System dynamics modeling, Peak carbon, Scenario simulation

## Abstract

Urban passenger transportation, as a pivotal element of the transportation system, accounts for over 40% of total carbon emissions from road transport. Consequently, mitigating carbon emissions in this sector is a crucial strategy for attaining carbon peak targets. This study centers on Lanzhou, a representative transportation hub city in China, and develops a dynamic model based on the Passenger Urban Transportation Carbon Emission System (PCES) framework to simulate emissions under three categories of interventions: Demand, Management, and Technology (DMT). The investigation analyzes the temporal trends and underlying mechanisms influencing these emissions. Results reveal that total carbon emissions from passenger transportation in Lanzhou are projected to rise until 2030, with a marked deceleration in growth rate anticipated after 2028. The carbon reduction efficacy among different interventions varies significantly, with fuel vehicle restrictions and management policies demonstrating the greatest effectiveness in conserving energy and reducing emissions. Nevertheless, continuous technological innovation and strategic policy guidance remain indispensable, especially to enhance public transportation usage and reduce overall energy consumption. Furthermore, the integration of multiple strategies accelerates progress toward achieving the ‘carbon peak’ objective within the passenger transportation sector. Simulation outcomes from the combined DMT scenario exhibit superior explanatory power regarding carbon reduction effects within the PCES framework compared to individual measures. Moreover, this research substantiates the utility of the PCES framework in steering the low-carbon development pathway of urban passenger transportation.

**Supplementary Information:**

The online version contains supplementary material available at 10.1186/s13021-025-00343-y.

## Introduction

Data from the International Energy Agency (IEA) reveals that the transportation sector accounts for approximately 23% of global carbon emissions. Within this sector, urban passenger transportation—including both public and private modes—constitutes the largest share of emissions, thereby significantly contributing to global climate change (Li and [35, 36]. To address climate change and advance sustainable development goals, international emission reduction frameworks such as the Kyoto Protocol and the Paris Agreement have been established [47]. Numerous countries have delineated specific targets and measures to curtail carbon emissions in transportation within their Nationally Determined Contributions (NDCs). These measures encompass the promotion of electric vehicles, enhancement of public transit systems, and improvement of fuel efficiency. The European Union has introduced policies to accelerate electric vehicle adoption, develop clean fuels, optimize urban transportation planning, and phase out internal combustion engine vehicles [52] Additionally, nations such as Sweden, Norway, and Finland have implemented transportation-focused carbon taxes aimed at reducing fossil fuel consumption by increasing operational costs and incentivizing clean energy uptake [61] As the world’s largest automobile market, China actively enforces regulatory frameworks targeting carbon emissions in urban transportation, including fuel consumption standards, new energy vehicle regulations, and carbon sequestration programs. Incentive mechanisms employing both rewards and penalties are utilized to stimulate the production and adoption of low-emission vehicles.

The rapid acceleration of urbanization, coupled with significant growth in urban populations, has driven a substantial surge in demand for urban passenger transportation, leading to a persistent increase in carbon emissions from the transportation system. This trend is particularly pronounced in developing countries, where rapid urbanization and motorization have directly fueled sharp rises in carbon emissions [45]. According to data from the Ministry of Public Security of China (2023), the number of motor vehicles in China has reached an unprecedented 430 million, with passenger cars accounting for 76.74%. Notably, the total number of licensed motor vehicle drivers in China stands at 520 million, surpassing the number of registered vehicles [36]. Carbon emissions from urban passenger transportation represent over 40% of total road transportation emissions, placing enormous pressure on urban transport systems and posing significant challenges to sustainable urban mobility. To confront these challenges, the Chinese government issued the "Comprehensive Work Plan for Energy Conservation and Emission Reduction during the 14th Five-Year Plan" in January 2022, mandating that carbon emissions within the transportation sector achieve internationally advanced standards [46]. The "Carbon Peaking Action Plan before 2030" further stipulates a target to reduce the carbon emission intensity of operational transportation units, measured by turnover, by approximately 9.5% relative to 2020 levels by 2030. Currently, the majority of Chinese cities are undergoing development and transition into medium and large urban centers, with population travel demands becoming increasingly diverse and extensive, thereby complicating efforts to meet carbon peaking objectives. Carbon emissions from urban passenger transportation are closely linked to vehicle quantity and travel frequency but are also substantially influenced by urban transportation infrastructure, travel behavior, and related policy frameworks.

Extensive research has been conducted to identify determinants of transportation carbon emissions and evaluate the environmental impact of urban passenger transportation, considering factors such as population size, GDP, energy intensity, and vehicle ownership in assessing potential emission reduction benefits [49]. Nevertheless, the determinants of carbon emissions in urban passenger transportation are complex and multifaceted, shaped by urbanization processes, population density, income levels, and travel demand, while constrained by geographic characteristics, energy mix, and policy orientations. In recent years, the Chinese government has vigorously promoted technological innovations within the transportation sector, fostering widespread adoption of new energy vehicles and encouraging public transit, particularly through the deployment of electric vehicles, intelligent transportation systems, and shared mobility technologies, which have collectively contributed to carbon emission reductions [14]. Moreover, policy interventions exert influence by guiding social behaviors and technological applications, forming an interconnected framework wherein demand management and technology mutually reinforce one another. Accordingly, comprehensive analysis of demand-side, management, and technological measures is essential for accurately assessing carbon emission trends in urban passenger transportation. This integrative approach provides a practical foundation for formulating effective emission mitigation strategies and advancing sustainable urban development.

Existing models for assessing urban transportation carbon emissions primarily include the Low Emission Urban Transportation (LEUT) model [23], the Multi-Region Input–Output (MRIO) model [71], and the Intercity Passenger Transportation Carbon Emissions (CE) model [64]. While these models offer valuable insights into transportation-related emissions, they predominantly adopt macroeconomic perspectives or focus on isolated policy scenarios, typically relying on static or linear assumptions [79]. Consequently, they are limited in capturing the complex, coupled interactions among urban passenger travel demand, policy interventions, and technological development. Moreover, insufficient consideration of variations in travel behavior and policy environments across specific urban contexts constrains their applicability for localized policy-making. Addressing these limitations, system dynamics (SD) methodologies have gained prominence in transportation carbon emissions research due to their capacity to model feedback loops and dynamic evolution across multiple interconnected components. This approach has evolved from analyzing isolated elements and strategies to comprehensive frameworks integrating multiple factors and interventions [60]. For instance, [8] employed an SD model to evaluate energy consumption and emission changes under supply- and demand-side policies in Istanbul, demonstrating the critical influence of policy coordination on emission reduction outcomes [8] Similarly, [70] utilized SD scenario analysis to examine the evolution of energy use and carbon emissions in Beijing’s passenger transport sector under various public transport prioritization strategies, highlighting the pivotal role of public transit development in facilitating a low-carbon transition [70]. Additionally, an SD decision-making model centered on transit-oriented development (TOD) emphasized that curbing private car usage constitutes a key pathway to achieving low-carbon targets [17]. Despite incorporating feedback mechanisms and dynamic evolution, these studies lack a systematic representation of the integrated effects of multi-dimensional factors—including demand, management, and technology (DMT)—and inadequately address the influence of heterogeneous passenger perceptions regarding cost and accessibility on emission trajectories. To overcome these gaps, the present study introduces the Urban Passenger Transport Carbon Emission System (PCES) model, which integrates demand-management-technology (DMT) strategies within the SD framework and extends the STIRPAT model [76, 57]. Using Lanzhou—a prototypical transportation hub city in China—as a case study, the model simulates carbon emission trajectories across 19 distinct scenarios. This approach enhances adaptability to dynamic feedback and scenario analysis, systematically elucidates the synergistic effects of combined strategies on emission reduction, and supports the development of informed, effective urban low-carbon transportation policies.

This study investigates the effectiveness of DMT measures in reducing carbon emissions from urban passenger transportation systems under multiple scenario conditions, as well as their performance across varying contexts. Utilizing multi-source data, a PCES framework is developed to quantify and analyze the collective emission reduction impact of DMT interventions and to formulate targeted policy recommendations. Moving beyond the validation of a singular theoretical hypothesis, the study systematically explores and substantiates integrated solutions aimed at achieving urban carbon reduction targets. Employing a system dynamics model, the research simulates urban passenger transportation carbon emissions across diverse scenarios, thereby offering scenario-driven decision-support for policymaking and practical guidance tailored to Lanzhou. The primary contributions of this study include:

1.  The development of a system dynamics model (PCES) for urban passenger transportation that integrates DMT measures;
2.  The identification of CO_2_ reduction potentials and underlying mechanisms of DMT strategies through comparative analysis of 19 distinct scenarios; and
3.  The validation of the model’s applicability and policy feasibility using Lanzhou, a representative transportation hub city, as a case study.

## Literature review of theoretical methods

Recent research has extensively examined the causes, impacts, and mitigation strategies of carbon emissions from urban passenger transportation, aiming to establish a robust scientific basis for emission reduction and sustainable urban development. The determinants of carbon emissions in this sector are complex and multifaceted, spanning social, economic, and environmental dimensions. Foundational theoretical frameworks such as the IPAT model [13], [76] and the transportation energy demand model have elucidated the relationship between carbon emissions and transportation systems [72]. Investigations by [24, 29] highlighted the significant contribution of passenger vehicles to urban greenhouse gas emissions and examined the influence of urban design on these emissions [24, 29]. Studies focusing on China’s urban passenger transportation sector reveal that optimizing street networks and urban planning presents considerable potential for carbon emission reductions [22, 62]. Nonetheless, effective strategies must account for regional variations in economic development, spatial environments, and mobility patterns [34, 56]. Complementing this, research on spatial organization’s role in CO_2_ emissions advocates infrastructural interventions to reduce mobility demands, facilitating transitions toward low-carbon economies [67]. Further analyses explore the nuanced relationships between transportation modes and emissions within urban contexts. For example, a Finnish transportation survey by [50] demonstrated that greenhouse gas emissions from air travel may offset vehicle emission reductions in densely populated cities [50, 69] developed a comprehensive estimation method incorporating cars, rail transit, taxis, and buses, identifying bus travel as the most energy-efficient mode. Additionally, measures such as transitioning to alternative fuels, improving vehicle fuel efficiency, and encouraging public transit use are effective in achieving short-term CO_2_ emission reductions within the transportation sector [37]. Dynamic vehicle restriction policies also play a vital role, [73] demonstrated that carbon emission taxes can influence passenger demand, modal choice, and traffic flow, thereby alleviating congestion and pollution [73]. Similarly, Batur et al. [8] applied a system dynamics model to Istanbul, assessing the impacts of supply- and demand-side policies on energy consumption and CO_2_ emissions in urban passenger transportation [8] In Qatar, [4]emphasized sustainable urban transportation through initiatives like the Doha Metro project, aiming to curtail emissions from private vehicles [4]. Collectively, these studies underscore the critical roles of travel demand management, policy enforcement, and technological innovation in reducing urban passenger transportation carbon emissions and advancing sustainable urban development.

Urban transportation carbon emission modeling constitutes a complex and pivotal research domain aimed at accurately predicting and managing transportation-related carbon outputs. Pan et al. [51] conducted a comparative analysis of CO_2_ emissions between new energy buses and conventional diesel buses across multiple regions, underscoring the critical need for precise estimation methodologies [31, 38] developed a multi-objective optimization model addressing urban passenger transportation structures that integrates low-carbon priorities alongside diverse stakeholder interests [38, 81] performed scenario-based analyses of energy consumption and emissions within the transportation sector, illustrating the emission reduction potential achievable through optimized transportation infrastructure [81]. The increasing application of big data analytics and machine learning techniques for forecasting transportation carbon emissions has been documented in recent studies [10], demonstrating that advanced computational models can effectively assess road transport carbon footprints. Additionally, uncertainty inherent in carbon emission estimates has been addressed and validated via rigorous model testing [9, 11]. System dynamics (SD) approaches have emerged as powerful tools to unravel the complex drivers of transportation carbon emissions by capturing feedback loops and interactions among various factors [16, 20]. For example, [68] proposed an integrated model combining multi-agent systems with system dynamics to simulate policy impacts on urban transportation CO_2_ emissions through job-housing balance adjustments [20]. Furthermore, [55] utilized an SD decision-making model centered on TOD to mitigate transportation sector emissions, emphasizing the critical role of reducing private car usage in achieving carbon reduction targets [42, 42] assessed the vulnerability of dynamic vehicle restriction policies during severe air pollution episodes, highlighting the significant efficacy of policy interventions in mitigating environmental risks [42].

The urban passenger transportation carbon emission system constitutes a complex, nonlinear framework characterized by significant variability in contributions from diverse transportation modes and traffic management measures, complicating accurate quantification of associated CO_2_ emissions. Existing studies identify three primary drivers—social demand, policy environment, and technological progress—as direct determinants of carbon emissions in urban passenger transportation [1, 5, 15]. Foundational research has elucidated structural emission characteristics and policy potentials, exemplified by Mogridge’s [48] transportation-induced demand hypothesis, which highlights a feedback loop between infrastructure expansion and trip growth [48], and Banister’s (1995) sustainable transportation assessment framework emphasizing the synergistic effects of modal shifts and energy efficiency improvements [7]. Recent projections by Maaouane (2022) and Sun (2023) model transportation energy demand in developing countries under varied policy scenarios [44, 63], Ribeiro and Mendes [54] explored zero CO_2_ emission strategies for public transit in Portugal through near- and medium-term technology scenarios based on varying assumptions [54]. The interactions among these measures display dynamic nonlinear characteristics, presenting significant challenges for traditional static analytical methods to accurately capture their complexity [59]. Scenario simulation analysis provides an innovative approach to overcoming this limitation and has been effectively applied in the urban transportation sector [65, 66]. For example, [21] assessed the environmental benefits of time-dependent green routes in the Greater Buffalo-Niagara region [21], while. [40] employed system dynamics scenarios to analyze energy consumption and CO_2_ emissions in Beijing’s urban passenger transportation, underscoring the critical role of public transit prioritization [40] Aggarwal and Jain [2] simulated integrated policy interventions to forecast energy demand and CO_2_ emissions for urban road transportation in Delhi by 2021 [2]. A scenario-based study of future policies impacting highway passenger transport and work-life balance in the Beijing-Tianjin-Hebei region demonstrated that reliance solely on public transportation is insufficient to curb urban energy consumption and carbon emissions in developing countries,effective mitigation also requires advancements in clean power generation and technology [43].

The development of low-carbon passenger transportation infrastructure exhibits significant regional and urban disparities, driven by variations in economic development and urbanization levels. Key factors such as public transit expansion, electrification of transport modes, and the effectiveness of carbon reduction policies frequently face challenges related to policy flexibility, public acceptance, and technological maturity [80]. Building on existing research, this study employs a system dynamics model to forecast urban passenger transportation carbon emission trends by incorporating residents’ travel demand, policy interventions, and technological progress. It proposes a holistic strategy to mitigate emissions while addressing the rising travel demands of urban populations, aiming to achieve carbon emission peaks in passenger transportation by 2030 and thereby advance China’s low-carbon urban development goals.

**Fig. 1:**
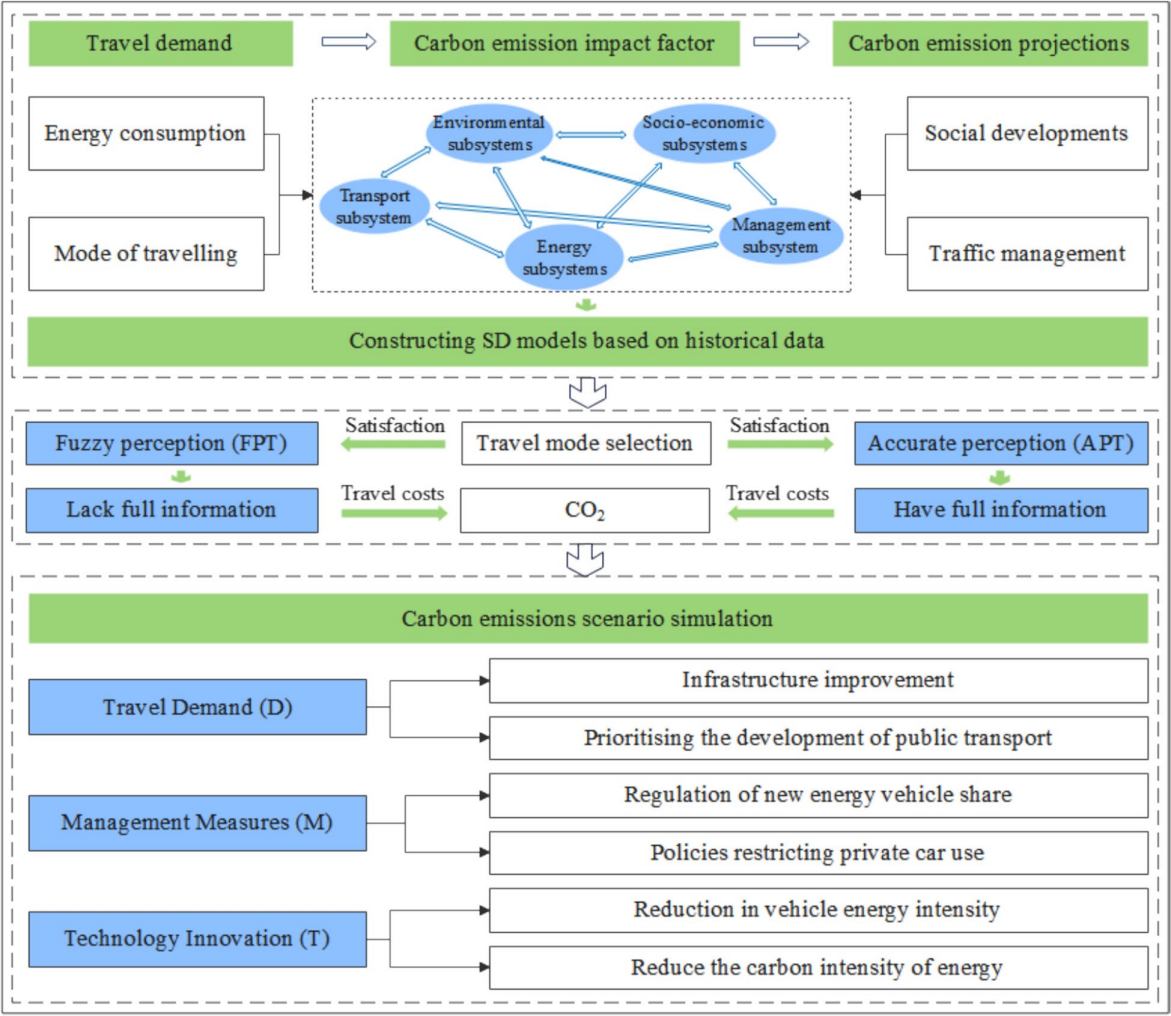
Dynamic framework for urban passenger transport carbon emissions based on scenario awareness and DMT

Despite considerable advances in urban transportation carbon emission modeling—particularly in establishing emission accounting frameworks and assessing policy scenarios—several limitations constrain their applicability and explanatory capacity in complex systems analysis and policy support. First, most models rely on static or linear structures, impeding accurate representation of dynamic feedback loops among policy regulation, behavioral adaptation, and technological innovation, as well as the nonlinear interactions shaping CO_2_ emission trajectories. Second, these models often exhibit limited integration of multiple influencing factors, typically analyzing single dimensions in isolation, and thus fail to systematically capture the synergistic effects of demand management, supply regulation, and technological transformation. Third, many frameworks lack localization tailored to specific urban spatial, structural, and governance contexts, undermining their transferability and operational relevance in localized policy design. Consequently, there is an imperative to develop a comprehensive assessment framework characterized by dynamic responsiveness, multi-factor integration, and local adaptability. Such a framework would effectively model the systemic evolution of urban passenger transportation carbon emissions across multiple scenarios, providing theoretical foundations and simulation tools to guide differentiated emission reduction pathways and the formulation of integrated policy solutions Figure 1.

## Materials and methods

This paper develops a comprehensive research framework centered on the feedback mechanisms within the PCES, incorporating behavioral perception heterogeneity and scenario-based simulations. Grounded in system dynamics theory, the framework integrates transportation, energy, socio-economic, and management components to construct a multi-subsystem coupled feedback model that dynamically captures the causal pathways driving urban passenger transportation carbon emissions. To account for heterogeneity in residents’ travel behavior regarding cost sensitivity and accessibility, the framework introduces “accurate perception (APT)” and “fuzzy perception (FPT)” scenarios, reflecting the potential influence of travel structure variations on CO_2_ emissions. Subsequently, these scenarios are combined to simulate emission reduction effects across three core pathways—travel demand, management, and technology—enhancing the model’s explanatory power through dynamic behavioral feedback and providing a robust analytical tool for scenario development and mechanism identification in the low-carbon transition of urban transportation under multiple policy initiatives.

### Regional overview

**Fig. 2:**
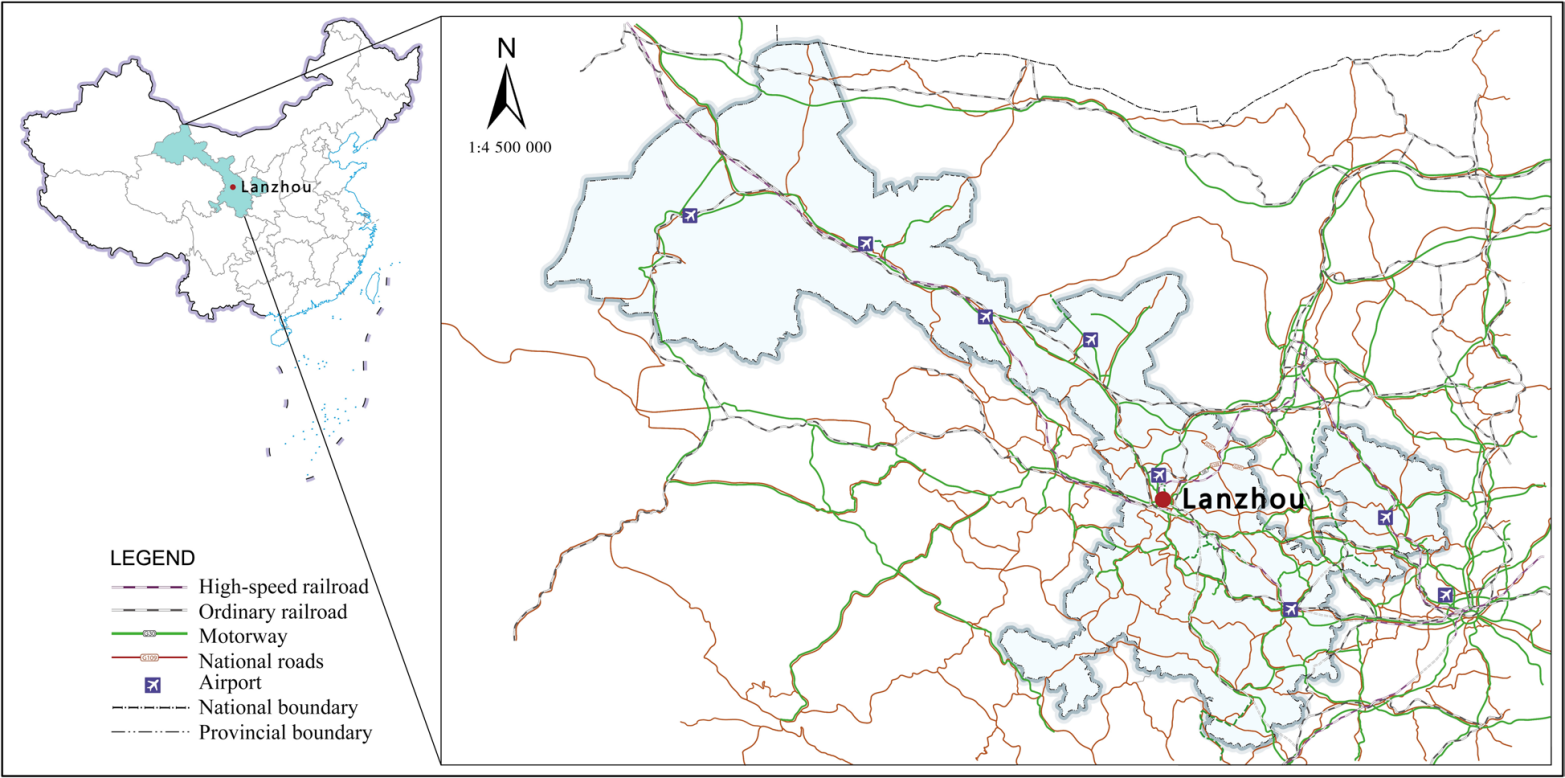
Study area

Lanzhou, located at the geographic center of mainland China, occupies a strategic position as the “Six Links in the Center” within northwest China. It serves as a critical node on the Silk Road Economic Belt and functions as a major national hub for energy and raw material industries [39]. The city hosts 12 national-level comprehensive trunk transportation hubs spanning rail, highway, and aviation networks, alongside the region’s second-largest international dry port, integrating freight distribution and container transfer centers (Fig. 2). Under the “Belt and Road” initiative, Lanzhou has effectively balanced developmental demands with ecological sustainability, achieving substantial economic growth. The city’s GDP and residents’ income have expanded at rates exceeding national averages. According to the latest Lanzhou City National Economic and Social Development Statistics Bulletin, the permanent population stands at approximately 4.436 million—an increase of 11,400 compared to the previous year—with regional GDP reaching 374.23 billion yuan, reflecting a 5.0% year-on-year increase. Per capita regional GDP rose by 4.7% to 84,460 yuan. These updated socioeconomic indicators provide a realistic context for the study area, while the model simulations are based on historical data spanning 2011 to 2022.

### Datasets

This study primarily utilized publicly available government data as its data source. Information on energy consumption, motor vehicle ownership, and Gross Regional Product (GRP) for the region was obtained from statistical yearbooks covering 2011 to 2022, including the China Statistical Yearbook and the China Energy Statistical Yearbook. Socio-economic and energy consumption data were mainly drawn from official statistics, policy documents, and development plans published by the Lanzhou municipal government. Key reference materials included the Gansu Provincial National Economic and Social Development Statistics Bulletin, the 2011–2022 Lanzhou National Economic and Social Development Statistics Bulletin, the Gansu Programme for a Stronger Transportation Nation, the Lanzhou 14th Five-Year Plan, and the Gansu Provincial Carbon Peak Implementation Programme. Residual uncertainties in the data were systematically classified, calibrated, and statistically processed by comprehensive comparison and analysis to form the research dataset. Notably, carbon emissions data from passenger transport during 2020–2022 exhibited significant anomalies due to COVID-19 travel restrictions. Consequently, only data from 2011 to 2019 were employed for model validation in Sect. 4.2 to ensure robustness. Pandemic-period data were incorporated into subsequent scenario simulations using a time-weighted smoothing approach to mitigate the influence of abnormal fluctuations on predictive outcomes.

### Energy consumption and carbon emission statistics

Lanzhou City’s total energy consumption demonstrated rapid growth between 2010 and 2022. Since 2015, external factors such as energy structure adjustments have notably influenced the city’s carbon emission levels. As a major energy hub sourcing supplies primarily from Xinjiang and the Gansu Hexi region, Lanzhou plays a critical role in Xinjiang coal exports and the West–East gas and power transmission network, linking energy consumption closely to shifts in the energy structure. Over the past decade, Lanzhou has experienced two significant fluctuations in carbon emission growth rates, largely attributable to industrial transformation and energy structural changes, resulting in considerable variability in energy demand. The city’s carbon emission intensity reduction target during the 13th Five-Year Plan was set at 17%. According to 2020 statistics, Lanzhou’s Gross National Product (GNP) reached 277.573 billion yuan, with total carbon emissions amounting to 66.5136 million tonnes and a carbon emission intensity of 2.40. Over the past ten years, total carbon emissions decreased by 24.43%, exceeding the designated reduction target (Table 1).

**Table 1:** Statistics on population, GDP and carbon emissions of Lanzhou between 2010–2022

Time (Year)	Population (million)	Carbon emissions (million tonnes)	GDP (billion yuan)	Carbon emission intensity (t/10^4^ yuan)
2010	3.619	39.39205	112.959	3.49
2011	3.687	39.55098	139.166	2.84
2012	3.771	37.32237	161.316	2.31
2013	3.834	46.17519	181.024	2.55
2014	3.906	49.24116	197.777	2.49
2015	3.979	40.54314	210.225	1.93
2016	4.057	35.22268	220.742	1.60
2017	4.143	38.63226	244.508	1.58
2018	4.215	40.09362	273.294	1.47
2019	4.287	42.10932	283.736	1.48
2020	4.372	66.51360	277.573	2.40
2021	4.384	69.70560	323.129	2.16
2022	4.415	71.88525	334.350	2.15

### Methods

#### Carbon emission measurement models for passenger transportation

The Greenhouse Gas Inventory Guidelines issued by the United Nations Intergovernmental Panel on Climate Change (IPCC) and other authoritative organizations provide standardized carbon emission factors for the transportation sector. According to the 2019 refinement of the 2006 IPCC Guidelines for National Greenhouse Gas Inventories, direct emissions are estimated using both top-down and bottom-up methodologies [12]. Carbon accounting is anchored to the geographical boundaries of the relevant country or region. Key parameters in the calculation include the number of transportation modes, travel distances, unit energy consumption, and carbon emission coefficients. The carbon emission calculation follows the formula below:

1 $$C = \sum\limits_{i = 1}^{5} {C_{i} }$$

2 $$C_{i} = \sum\limits_{j = 1}^{n} {E_{ij} } \times r_{j}$$

3 $$E_{ij} = S_{i} \times M_{i} \times N_{ij}$$

where, ${\text{C}}$ is the total amount of CO_2_ generated by the passenger transportation sector; ${\text{C}}_{\text{i}}$ is the carbon emissions from the category ${\text{i}}$ motor vehicles in the passenger transportation sector; ${\text{E}}_{\text{ij}}$ is the fuel consumption of the category ${\text{i}}$ motor vehicles; ${\text{r}}_{\text{j}}$ is the carbon dioxide emission coefficient of the category ${\text{j}}$ energy sources, in unit of tonnes of carbon/trillion joules; ${\text{S}}_{\text{i}}$ is the number of categories ${\text{i}}$ motor vehicles; ${\text{M}}_{\text{i}}$ is the number of kilometres of category ${\text{i}}$ motor vehicles; ${\text{N}}_{\text{ij}}$ is the energy consumption per unit of category ${\text{i}}$ motor vehicles.

#### Extended STIRPAT model

This study draws on existing literature to categorize the key factors influencing the PCES framework. As detailed in Table 2, socio-economic factors include population size, economic development level, and infrastructure capacity; travel demand factors encompass transportation mode share, residents’ travel satisfaction, and passenger transport structure; policy factors cover service volume, traffic conditions, and regulatory measures; while energy intensity is classified as a technical factor. To model the interrelationships among these variables within the PCES framework, VENSIM software (Personal Learning Edition) was employed to extend the STIRPAT model, itself derived from the IPAT framework [31]. The calculation formula is presented below.

4 $$I = P \times A \times G \times T$$

where, ${\text{I}}$ is the environmental impact; ${\text{P}}$ is the socio-economic factor; ${\text{A}}$ is the travel demand; ${\text{G}}$ is the policy factor; ${\text{T}}$ is the technological factor.

**Table 2:** Classification and theoretical sources of each type of influencing factor

Form	Representative indicators	Definition of indicators	Literature sources
Socio-economic factors	Population	The resident population reflects the potential for transportation demand	[6, 1]
	GDP	GDP measures regional economic vitality and determines willingness and ability to travel	
	Transportation infrastructure	Road mileage, track length, etc. represent the load-bearing base of the transportation system	
Travel demand factors	Mode of transportation	The share of public transportation, private car and shared trips reflects mode choice preferences	[75, 69]
	Travel satisfaction	Travel satisfaction surveys reflect the quality of the travel experience	
	Passenger transportation structure	Sharing rates such as urban passenger bus and rail represent macro travel trends	
Policy factors	Passenger service volume	The total number of passengers and frequency of trips measure the level of government provision	[41, 18, 78]
	Traffic operation status	Road speed and congestion index reflect real-time operational efficiency	
	Policies and regulations	Incentive and restriction policies reflect the strength of institutional intervention	
Technical factor	Energy intensity	Energy intensity measures the level of technical efficiency of transportation	[27]

The classical IPAT model is primarily suited for qualitative analysis, limiting its applicability in quantitative contexts. To address this, researchers introduced stochastic elements, resulting in the evolution of the STIRPAT model, which enhances its suitability for analyzing the determinants of environmental impacts. The STIRPAT model is formulated as follows.

5 $$I = P \times A \times G \times T = aP^{b} A^{c} G^{d} T^{e} \delta$$

where $aa$ is the coefficient of the model; $bb$, $cc$, $dd$, and $ee$ are the driving indices of $PP$, $AA$, $GG$ and $TT$, respectively;$\delta\delta$ refers to the random error.

This study focused on carbon emissions in the transportation sector as the dependent variable within the STIRPAT model. The model was extended by incorporating additional factors, including the share of new energy vehicles, the proportion of public transportation usage, and investment in road infrastructure.

6 $${\text{C}}_{\text{u}}\text{=}{\text{a}}{\text{X}}_{1}^{{\gamma }_{1}}{\text{X}}_{2}^{{\gamma }_{2}}{\text{X}}_{3}^{{\gamma }_{3}}{\text{X}}_{4}^{{\gamma }_{4}}{\text{X}}_{5}^{{\gamma }_{5}}{\text{X}}_{6}^{{\gamma }_{6}}{\text{X}}_{7}^{{\gamma }_{7}}{\text{X}}_{8}^{{\gamma }_{8}}\delta$$

where ${\text{C}}_{\text{u}}$ is the total carbon emission of urban passenger transportation, unit: 10,000 tonnes; ${\text{X}}_{1}$ is the total regional population, unit: 10,000 people; ${\text{X}}_{2}$ is the per capita GDP, unit: yuan/person; ${\text{X}}_{3}$ is the energy intensity, unit: tonnes of standard coal/million yuan; ${\text{X}}_{4}$ is the number of motor vehicles, unit: vehicle; ${\text{X}}_{5}$ is the ratio of new energy vehicles, unit: %; ${\text{X}}_{6}$ is the ratio of public transportation vehicles, unit: %; ${\text{X}}_{7}$ is the mileage of the highway, unit: kilometer; ${\text{X}}_{8}$ is the investment in road construction, unit: million yuan. Investment in road construction, unit: RMB 10,000 yuan.

Logarithmic transformations are typically applied to both sides of the equation during model analysis to mitigate heteroskedasticity and enhance the precision of the model results.

7 $$\begin{aligned}{\text{ln}}{\text{C}}_{\text{u}}&={\text{lna}}\text{+}{\gamma }_{1}{\text{ln}}{\text{X}}_{1}\text{+}{\gamma }_{2}{\text{ln}}{\text{X}}_{2}\\&\text{+}{\gamma }_{3}{\text{ln}}{\text{X}}_{3}\text{+}{\gamma }_{4}{\text{ln}}{\text{X}}_{4}\text{+}{\gamma }_{5}{\text{ln}}{\text{X}}_{5}\\&+{\gamma }_{6}{\text{ln}}{\text{X}}_{6}\text{+}{\gamma }_{7}{\text{ln}}{\text{X}}_{7}\text{+}{\gamma }_{8}{{\text{ln}}{\text{X}}}_{8}\text{+}{\text{ln}}\delta\end{aligned}$$

**Fig. 3:**
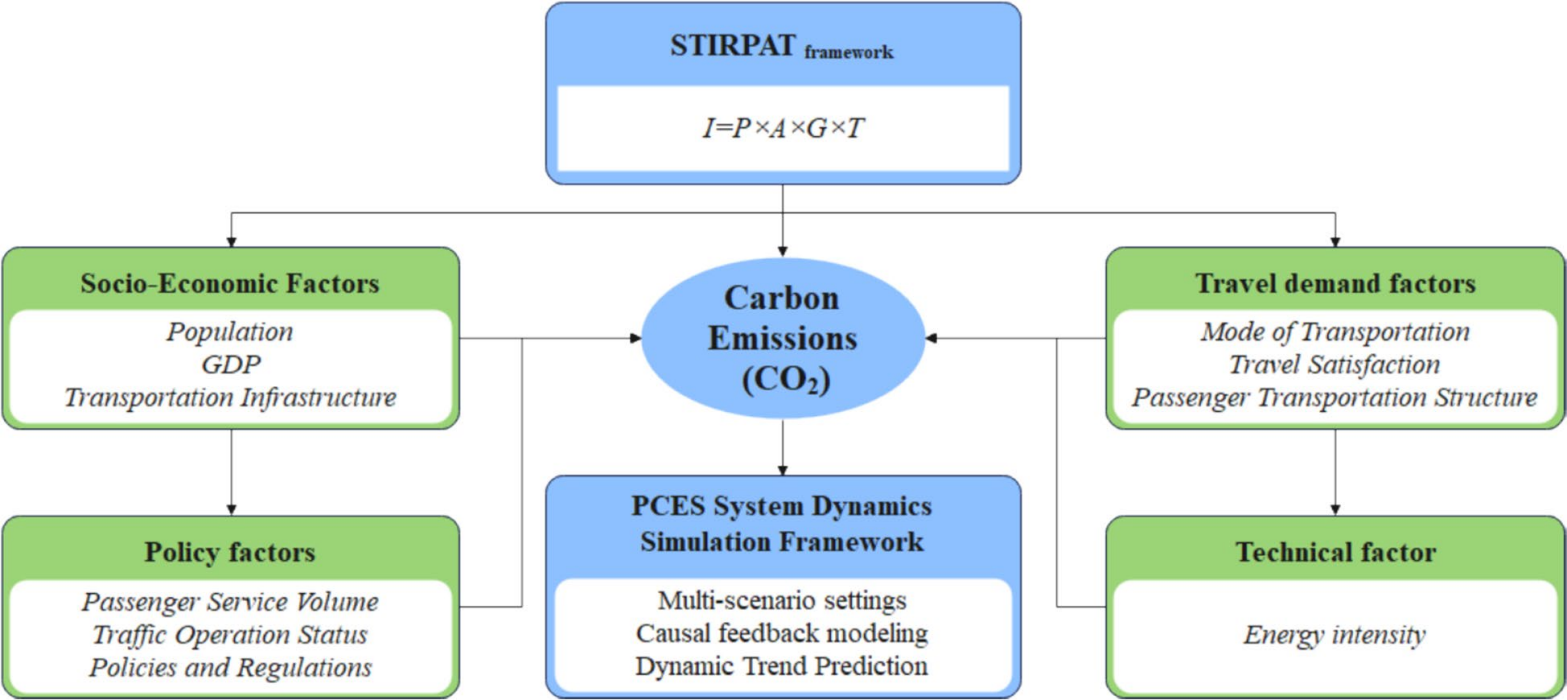
STIRPAT-PCES model fusion structure diagram

As illustrated in Fig. 3, the expanded STIRPAT model establishes a framework for identifying the principal drivers of CO_2_ emissions in urban transportation, forming the theoretical foundation for structuring variables within the PCES model. During system dynamics modeling, the core STIRPAT variables (P, A, G, T) are mapped onto four principal subsystems within the PCES framework, each contributing to the dynamic evolution of carbon emissions in the urban passenger transportation system. Building on this, the PCES model converts STIRPAT’s static structure into a dynamic feedback mechanism, enabling the simulation of multiple scenarios using Vensim.

To address multicollinearity in the STIRPAT model and its potential effect on results, ridge regression was employed. This technique enhances model robustness by introducing a non-negative regularization parameter, the ridge factor K, applied after logarithmic transformation of the equation [74, 35, 36, 53].

#### Model assumption

To construct the system flow diagram for the causal feedback system, several assumptions were made to streamline the model and exclude non-essential elements:

(1) The study exclusively targets carbon emissions from urban passenger transportation, deliberately omitting inter-city and long-distance modes such as air travel and inter-regional railways;

(2) Carbon emission calculations are standardized to CO_2_, accounting solely for fuel energy consumption during vehicle operation while disregarding energy use from equipment, facilities, and other fixed sources;

(3) For electric vehicles, emissions associated with electricity consumption are converted into equivalent standard carbon units for calculation consistency;

(4) Given the substantial disruptions to urban passenger transportation caused by COVID-19 during 2020–2022—characterized by abrupt declines in total passenger trips and shifts in travel mode structure—a time-weighted adjustment strategy was applied to mitigate short-term anomalies and avoid undue distortion of long-term structural modeling outcomes. This approach, adapted from established literature [19], involves smoothing sensitive variables such as traffic volume and modal share using an exponential weighted average method, with decay weights set at 0.8, 0.9, and 1.0 for the years 2020, 2021, and 2022, respectively. Widely adopted in system dynamics and econometric studies, this technique reduces bias introduced by anomalous periods while preserving the integrity of long-term trends. Validation comparing model outputs with and without this adjustment revealed differences in key simulation indicators within ± 5%, demonstrating that the method effectively attenuates pandemic-related distortions and enables the model to capture the resilience features of the passenger transport system during the crisis.

#### Causal loop and stock-and-flow diagram

**Fig. 4:**
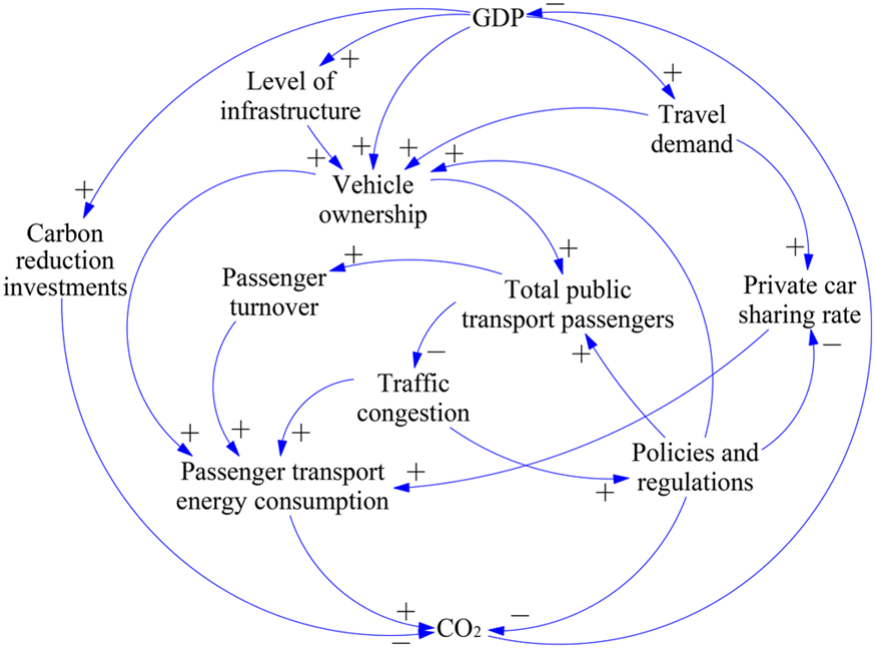
Causal loop feedback relationship diagram for PCES

The proposed SD model of passenger transportation carbon emissions integrates economic, transportation, travel demand, and energy consumption subsystems into a unified feedback framework. Socio-economic development, reflected by GDP growth, shapes residents’ travel demand and modal patterns, thereby driving the ongoing enhancement and expansion of the passenger transportation sector. Although advancements in passenger transportation improve travel convenience and stimulate rapid economic growth, changes in travel demand and structure exert a direct influence on overall energy consumption. Furthermore, the expanding passenger transportation industry may result in substantial energy use, with excessive emissions posing potential constraints on socio-economic progress. By examining the interdependent feedback mechanisms among these subsystems, a carbon emission feedback loop model for urban passenger transportation has been developed, with its principal feedback loops depicted in Fig. 4.

The system incorporates three primary feedback loops:

4. GDP → + Travel demand → + Private car sharing rate → + Passenger transportation energy consumption → + CO_2_ → – GDP;
5. ② GDP → + Infrastructure level → + Motor vehicle ownership → + Passenger turnover → + Passenger transportation energy consumption → + CO_2_ → – GDP;
6. ③ GDP → + Motor vehicle ownership → + Total public transportation passenger volume → – Traffic congestion → + Policies and regulations → + Private car sharing rate → + Passenger transportation energy consumption → + CO_2_ → – GDP.

**Fig. 5:**
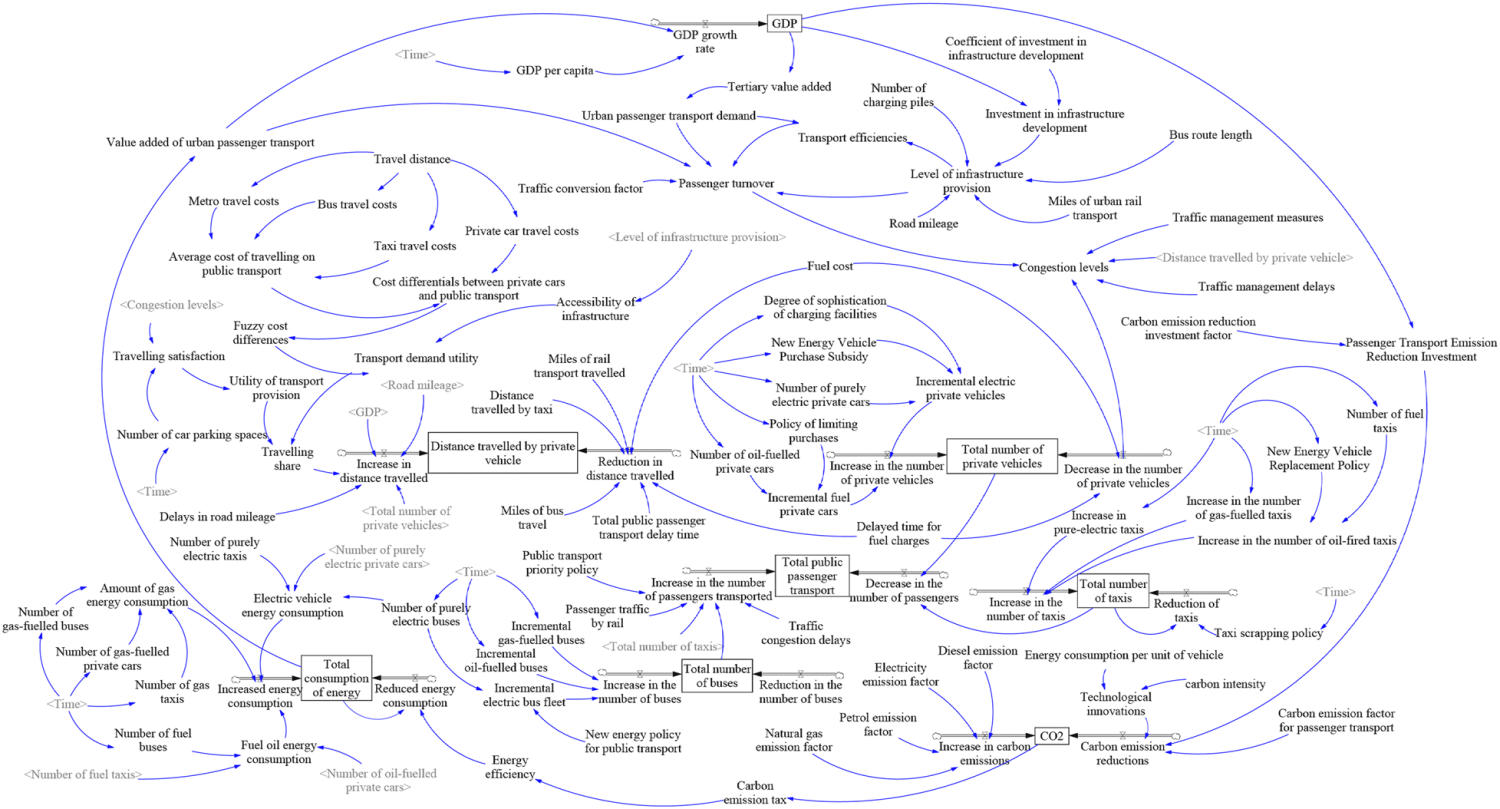
Causal loop diagram of the urban passenger transportation carbon emission system (PCES)

Figure 5 illustrates the causal loop diagram of the Passenger Carbon Emission System (PCES), integrating four interconnected subsystems: economic and infrastructure, passenger transport, travel demand and policy, and energy–carbon emissions. This model captures the dynamic interplay between passenger turnover and carbon emission intensity. The feedback loops demonstrate how GDP growth and infrastructure expansion stimulate passenger demand and modal shifts, affecting congestion, transportation efficiency, and energy consumption, thus shaping carbon emissions. Key mechanisms include the prioritization of public transportation, restrictions on private vehicle use, and advancements in energy technologies. Detailed variable definitions and the model’s 47 governing equations are provided in the Supplementary Materials.

### Scenario setting

Scenario design within this study is structured along three dimensions: residents’ travel demand, management measures, and technological advancements. Critical factors such as infrastructure development level, public transportation priority, new energy vehicle share, policy constraints, and energy and emission intensity are incorporated to establish key scenarios. Table 3 details comprehensive scenarios created by integrating multiple individual scenarios. Parameter settings are grounded in national and local “14th Five-Year Plan” transportation targets, historical trends from the Lanzhou Statistical Yearbook (2011–2022), and corroborated through reference to leading studies [33, 32, 26], ensuring alignment with realistic development trajectories. The comprehensive scenario (DMT) simulates synergistic emission reduction effects arising from combined strategies, reflecting plausible multi-strategy pathways in practice.

**Table 3:** Carbon scenario setting for urban passenger transportation

Scenario dimensions	specific programmes	scenario setting
Travel demand	Improvement of infrastructure development	ICL 1: Continuously increase the total mileage of urban roads to a growth rate of 10 per cent and increase investment in infrastructure by 10 per cent annually ICL 2: Continuously increase the total number of charging piles to reach a growth rate of 15 per cent ICL 3: Improve traffic management and control and further improve road access to reduce traffic congestion and its impact by 20 percent
	Prioritising the development of public transportation	PTP 1: 5 per cent annual increase in the number of buses and 5 per cent annual increase in the number of taxis PTP 2: 20 per cent increase in total public passenger transportation PTP 3: 5 per cent annual growth in the bus network
Management measures	Regulating the proportion of new energy vehicles	NVP 1: Increase the subsidy for new energy vehicle purchases and increase the share of new energy vehicles in private cars by 20 per cent NVP 2: Implement a new energy replacement policy for buses and taxis so that 10 percent of buses and 10 percent of taxis are replaced by new energy vehicles yearly
	Policies to restrict private car traffic, limit the number of vehicles and control fuel costs	RPR 1: Limits on the number or rate of growth of fuel private cars, which could be imposed by a policy of limiting purchases to a 10 per cent reduction in the annual growth rate RPR 2: Limits on the number of trips or distance travelled by private cars, which could be implemented by a number restriction policy that reduces their average annual distance travelled by 10 per cent and increases the average annual mileage travelled by taxis and buses by 5 per cent; and RPR 3: a linear increase in fuel costs; and RPR 4: Carbon tax grows to 50 yuan/tonne
Technology innovation	Reduced energy consumption per unit of vehicle	EII 1: Development of traditional vehicle technology to reduce energy consumption per unit by 5 per cent for petrol vehicles and 10 per cent for diesel vehicles; EII 2: Improvement of new energy vehicle technology, resulting in a 15 per cent reduction in unit energy consumption for electric vehicles
	Reducing the carbon intensity of energy	EIR 1: Development of energy technologies and upgrading of fuel quality, resulting in a 10 per cent reduction in the carbon emission factor for petrol and a 10 per cent reduction in the carbon emission factor for diesel EIR 2: Reduction of indirect emissions from electricity production, resulting in a 10 per cent reduction in the carbon emission factor for electricity
Combination of demand-management-technical measures		DMT 1: PTP1 + RPR1; DMT 2: NVP1 + RPR2 + EII2; DMT 3: PTP2 + RPR1 + EIR1 + EII1

ICL refers to Infrastructure Construction Level, PTP to Public Transportation Priority, RPR to Restriction Policy Ratio, NVP to New Energy Vehicle Promotion, EII to Energy Intensity Improvement, EIR to Emission Intensity Reduction, and DMT to integrated scenarios combining Demand, Management, and Technology measures

## Results

### Carbon emission accounting for passenger transportation in lanzhou

**Fig. 6:**
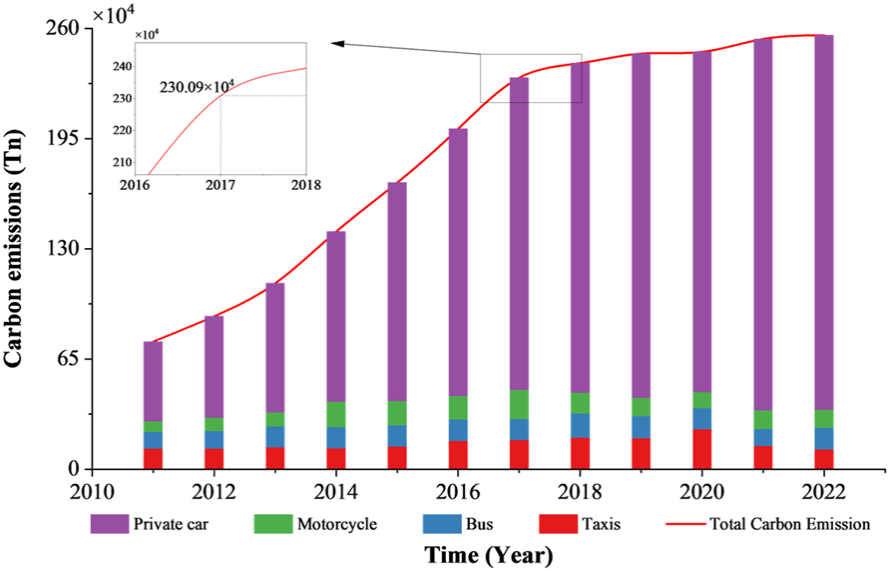
Trends in carbon emissions from different modes of transportation

Figure 6 depicts the evolution of carbon emissions from passenger transportation in Lanzhou over the past decade. Emissions increased from 0.752 million tonnes in 2011 to 2.309 million tonnes by 2017, reflecting a rapid upward trajectory aligned with Lanzhou’s accelerated economic and social development. However, post-2017, the growth rate of passenger transportation carbon emissions slowed notably, primarily due to shifts in residents’ travel behaviors and rapid advancements in new energy transportation technologies. The introduction of high-speed rail, metro, and intercity rail systems has substantially transformed commuting patterns. Over the same decade, the number of private vehicles surged from 162,300 to 703,700—an approximate fourfold increase—positioning private cars as the dominant source of transportation-sector carbon emissions. Although new energy vehicles have seen increased adoption since 2017, their market share in Lanzhou remained modest at 1.53% of the total private vehicle fleet by the end of 2021, exerting limited impact on overall emission mitigation. Additionally, recent local initiatives such as the phased launch of the metro system, promotion of shared bicycles, and development of pedestrian-friendly infrastructure have contributed to reducing urban transportation carbon emissions.

### Carbon emission modeling test for passenger transportation based on historical data

The COVID-19 pandemic’s global disruption from 2020 to 2022 led to stringent travel restrictions, causing significant volatility in transportation-related emissions. To prevent distortion of model validation by these anomalous years, data spanning 2011 to 2019 were selected for testing. As summarized in Table 4, three representative indicators from core subsystems—GDP, passenger turnover, and passenger transportation carbon emissions—were employed to assess the system dynamics (SD) model’s performance. Results demonstrated close alignment between simulated and observed data, confirming strong model consistency. Specifically, during the 2011–2019 interval, the maximum deviation between simulated and actual carbon emissions was 7.8%, with an average deviation of 2.89%. These outcomes substantiate the SD model’s robustness and its efficacy in accurately capturing the dynamics of urban passenger transportation carbon emissions under typical, non-pandemic conditions.

**Table 4:** Practical consistency tests of dynamic models for passenger transportation systems

Year	GDP(billions yuan)			Passenger turnover (ten thousand person-kilometres)			Carbon emissions from passenger transportation (tonnes)		
	Realistic	Simulated	Error	Realistic	Simulated	error	Realistic	Simulated	Error
2011	1391.7	1394.3	−0.20%	331,491	352,910	−6.50%	752,241	763,455	−1.50%
2012	1613.2	1641.9	−1.80%	481,165	509,716	−5.90%	901,898	972,660	−7.80%
2013	1810.2	1887.4	−4.30%	544,721	551,480	−1.20%	1,096,212	1,128,340	−2.90%
2014	1977.8	2006.4	−1.40%	582,882	613,667	−5.30%	1,401,791	1,359,963	3.00%
2015	2102.3	2215.1	−5.40%	626,095	647,852	−3.40%	1,690,123	1,709,144	−1.10%
2016	2207.4	2286.5	−3.60%	660,459	695,530	−5.30%	2,008,170	2,105,269	−4.80%
2017	2445.1	2358.2	3.50%	699,477	702,395	−0.40%	2,309,090	2,381,950	−3.2%
2018	2660.2	2766.4	−4.00%	704,450	691,420	1.80%	2,395,324	2,423,670	−1.20%
2019	2852.5	2970.9	−4.20%	429,249	466,235	−8.60%	2,450,960	2,438,156	0.50%

### Decomposition of carbon emission drivers for passenger transportation in lanzhou

**Fig. 7:**
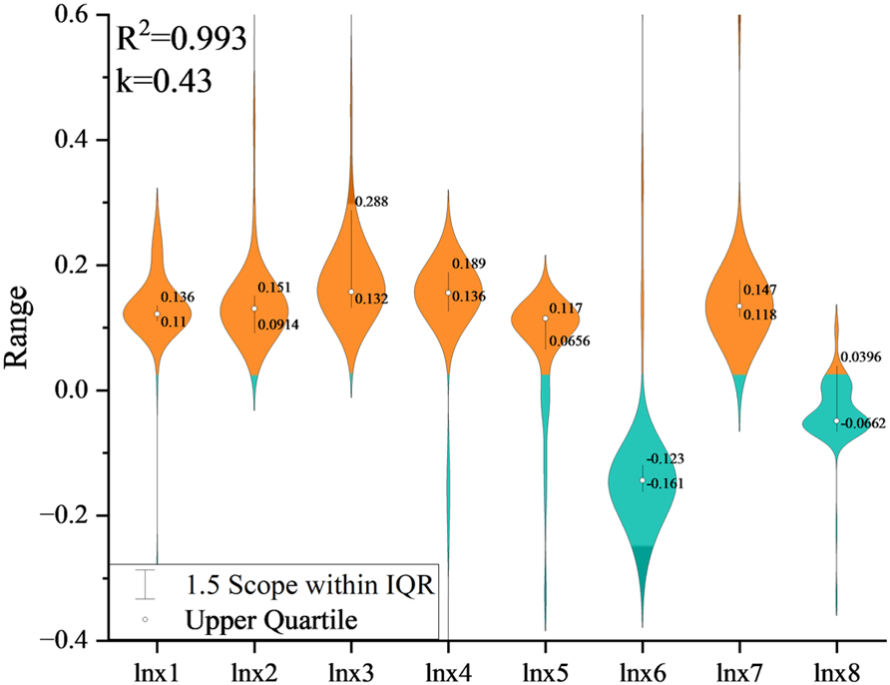
Carbon emission factors for urban passenger transportation by different modes of transportation

Figure 7 presents the ridge regression model employed to quantify the contributions of eight variables to carbon emissions from passenger transportation in Lanzhou. These variables include population size, per capita GDP, energy intensity, motor vehicle ownership, the share of new energy vehicles, public transportation vehicle count, road mileage, and investment in road infrastructure. The regression curve stabilizes at a ridge parameter of k = 0.43, yielding a coefficient of determination (R^2^) of 0.993.

Utilizing this optimal k value where R^2^ smooths, the ridge regression equation was derived to estimate the coefficients associated with each factor influencing urban passenger transportation carbon emissions.

8 $$\begin{aligned}C_{u} &= 15.44X_{1}^{0.123} X_{2}^{0.134} X_{3}^{0.159} X_{4}^{0.158} X_{5}^{0.115} X_{6}^{ - 0.145} X_{7}^{0.137} X_{8}^{ - 0.051}\end{aligned}$$

Regression analysis reveals positive correlations between carbon emissions and population size, per capita GDP, energy intensity, private car ownership, new energy vehicle proportion, and road mileage. Specifically, a 1% increase in regional population size corresponds to a 0.123% rise in total urban transportation carbon emissions, while a 1% increase in the share of new energy vehicles is associated with a 0.115% increase in emissions. Notably, energy intensity and private vehicle ownership exhibit the strongest impact, emerging as principal drivers of emissions growth, which may critically shape future carbon emission trajectories. Despite ongoing promotion, new energy vehicles currently maintain low market penetration and exert a minimal effect on emissions. Conversely, expanding the public transportation fleet significantly mitigates passenger transportation carbon emissions: a 1% increase in public transportation vehicles reduces total urban passenger transportation emissions by 0.145%, translating to a 0.051% decrease in overall urban transportation emissions. These findings underscore the benefits of increasing public transit capacity or utilization rates for energy conservation and emission reduction in urban passenger transport. Model robustness was confirmed through dual validation involving ridge regression stability testing and system dynamics sensitivity analysis. Stability was achieved at k = 0.43 (R^2^ = 0.993), with conclusions remaining consistent across perturbation ranges and aligning with related literature [3, 70], indicating strong reliability of the model estimates.

### Carbon emission modeling for passenger transportation based on scenario awareness

#### Analysis of total carbon emissions under sensing scenarios

**Fig. 8:**
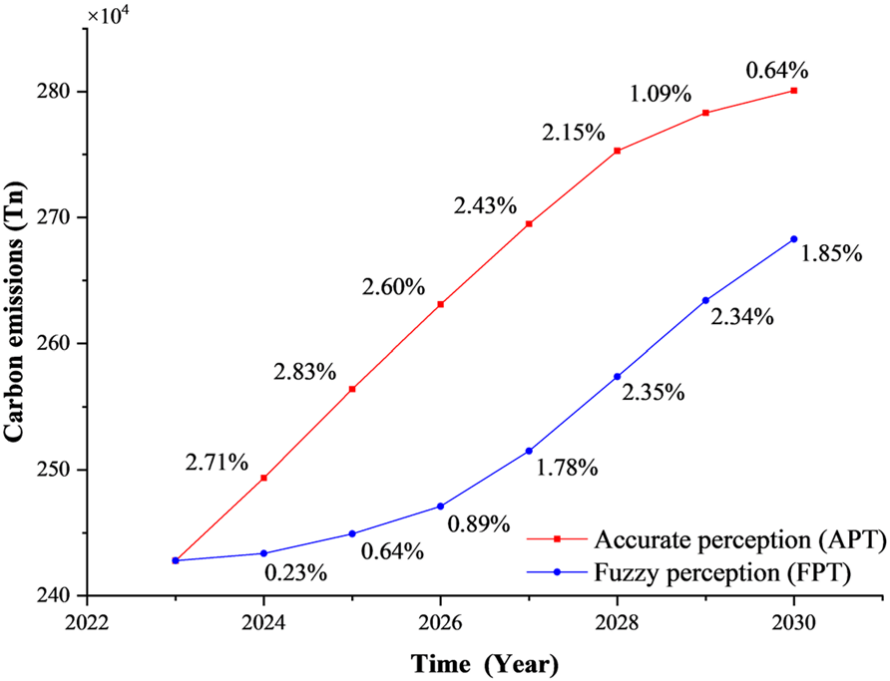
Trends in projected changes in carbon emissions

Urban residents’ travel mode choices are strongly influenced by differences in transportation costs and infrastructure accessibility. Fuzzy logic rules were applied to evaluate the demand and supply utilities guiding residents’ travel mode selection. As shown in Fig. 8, total carbon emissions from passenger transportation linked to residents’ travel modes were simulated under two demand scenarios: APT and FPT [77]. The results reveal a pronounced divergence in urban passenger transportation carbon emissions between these scenarios. Under the FPT scenario—where travel costs are disregarded—the annual growth rate of urban passenger carbon emissions gradually rises from 0.23% in 2023 to 1.85% in 2030, peaking at 2.35% in 2028 before gradually declining. Although emissions growth is evident in the short term, long-term trends indicate a deceleration attributable to saturated transportation demand and enhanced system efficiency. Conversely, the APT scenario, which accounts for travel costs and convenience, projects a rapid increase in passenger transportation emissions initially,however, following 2028, the average annual growth rate declines markedly from 2.15% to 0.64%, driven by improved acceptance of travel modes and new-energy public transportation. This scenario anticipates urban passenger transportation carbon emissions reaching a peak gradually after 2030, a projection corroborated by other studies [31, 70].

#### Analyzing carbon emissions under modeled scenarios

To project carbon emissions from urban passenger transportation under various future scenarios, sixteen scenarios were formulated based on three strategic dimensions: demand, management, and technology. These scenarios simulated total passenger transportation carbon emissions and were validated against previously obtained APT data.

(1) Analysis of total carbon emissions under demand scenarios.

**Fig. 9:**
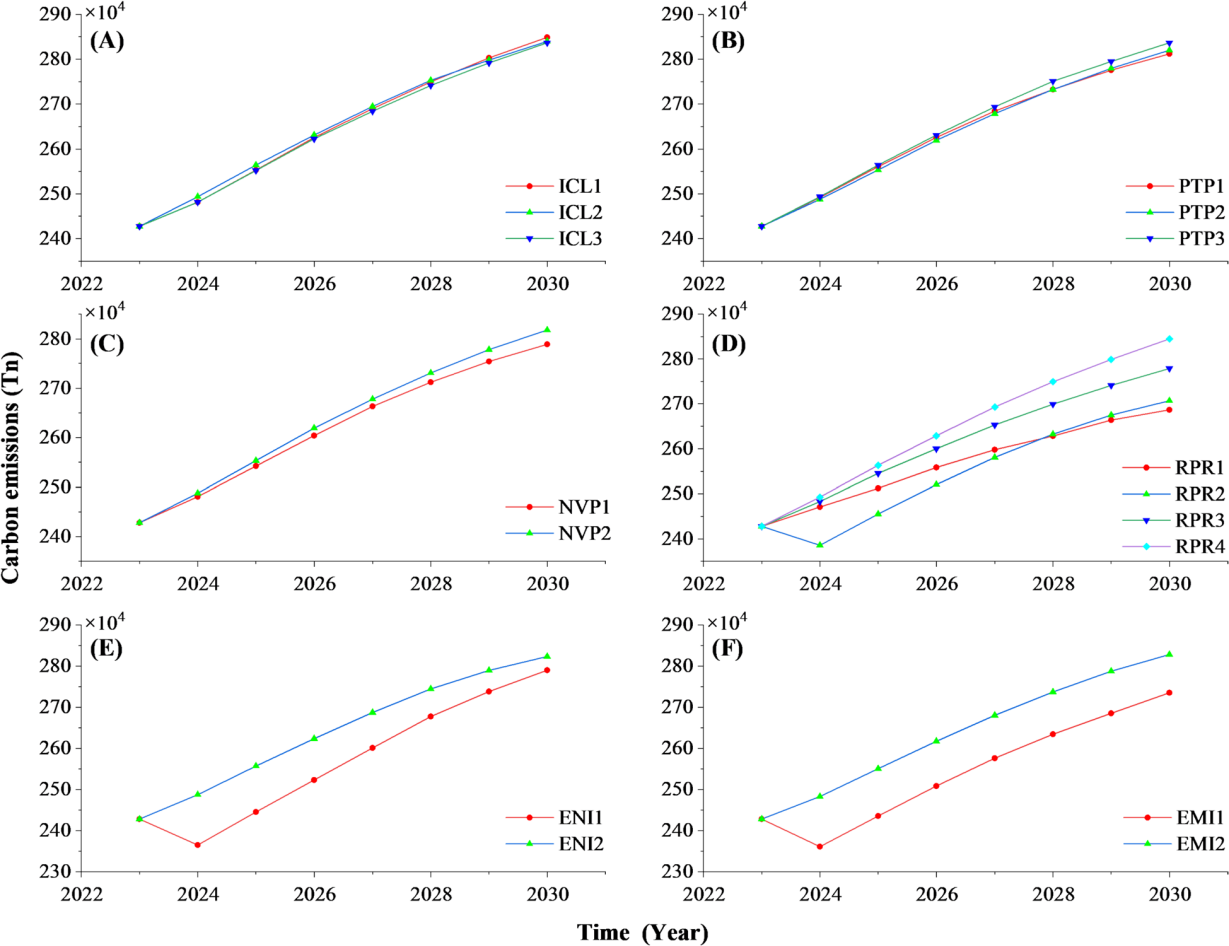
Trends in carbon emissions from passenger transportation under different scenarios

Simulation of three demand-driven scenarios—expanded road mileage, increased charging station availability, and alleviated traffic congestion in Lanzhou City from 2023 to 2030—indicated that infrastructure development exerts a relatively modest impact on carbon emissions from passenger transportation. Emission growth trends were comparable across scenarios, suggesting that while improved infrastructure can reduce congestion, it concurrently stimulates travel demand, increasing trip volumes across all modes. Consequently, the net effect of congestion mitigation on total carbon emissions remains limited (Fig. 9A). In contrast, scenarios prioritizing public transportation expansion—characterized by increased bus fleet size and passenger volume (PTP1, PTP2)—demonstrated effective carbon emission reductions. Notably, PTP1 surpassed PTP2 in carbon savings by 2028, with emissions reaching 273,239 tonnes, and by 2030, projected emissions under PTP1 amounted to 2.811 million tonnes, 0.8 million tonnes less than PTP2 and 24,900 tonnes less than PTP3. These outcomes imply that enhancing bus route coverage more effectively promotes transportation equity while reducing emissions. Compared to infrastructure improvements, prioritizing public transportation development yields a more substantial carbon reduction impact, achieving a maximum annual reduction of 27.2 thousand tonnes and an average annual decrease of 3.1 thousand tonnes, particularly after 2028. This underscores that optimizing public transit development to align with travel demand constitutes a more effective strategy for lowering passenger transportation carbon emissions (Fig. 9B).

(2) Analysis of total carbon emissions under policy guidance.

The rapid expansion of new energy vehicles is increasing their share within the overall motor vehicle fleet, aligning with anticipated trends in the passenger transportation industry. Across China, various regions have implemented subsidy programs encouraging the purchase of new energy vehicles for both private cars and buses. This study projected the carbon emission impacts of substituting traditional private cars with new energy private cars (NVP1) and new energy buses (NVP2) (Fig. 9(C)). Results indicate that a 20% increase in the market share of new energy private cars could reduce carbon emissions by 60.1 thousand tonnes per year relative to fuel-powered vehicles, lowering the emissions growth rate by 1.26%. Additionally, by 2030, emissions are projected to decline by 29.1 thousand tonnes per year compared to new energy buses (NVP2), largely because private cars outnumber buses, taxis, and other public transit modes. This suggests that increased adoption of new energy private cars effectively mitigates carbon emissions, consistent with China’s current promotion and subsidy policies. Conversely, regulatory measures targeting fuel vehicles—such as driving restrictions, purchase limits, fuel taxes, and carbon taxes—exert stronger influences on reducing emissions from passenger transportation. Evidence of this is seen under the RPR2 scenario, where total carbon emissions sharply decrease from 2.427 million tonnes in 2023 to 2.385 million tonnes in 2024 before gradually reverting to prior levels (Fig. 9(D)). This highlights the critical role of policy direction in managing carbon emissions in passenger transportation.

(3) Analysis of Total Carbon Emissions under Technological Innovation Scenarios.

Figure 9(E) and (F) illustrate traditional vehicle optimization strategies aimed at reducing per-vehicle energy consumption and improving fuel quality. While these conventional approaches yield modest emission reductions, their overall impact remains limited. In contrast, advancements in information technology—such as simulating new energy vehicles to reduce energy use and deploying wind and solar power to decrease indirect emissions—demonstrate substantially greater potential to curb future passenger transportation carbon emissions. Specifically, simulations of scenarios EII2 and EIR2 suggest that, relative to traditional technological innovations, these emerging technologies could lower total carbon emissions by an average of 81.6 thousand tonnes and 93.5 thousand tonnes per year, respectively, over the next decade. These findings underscore the pronounced benefits of promoting new energy vehicles and cutting-edge technologies for emission mitigation in transportation. Although technological innovations may induce demand fluctuations, their real-world deployment has been gradual and steady, indicating that such advancements are likely to contribute significantly to achieving carbon emission peaks in the transportation sector over the long term.

#### Total carbon emissions from passenger transportation in lanzhou city under scenario combination

**Fig. 10:**
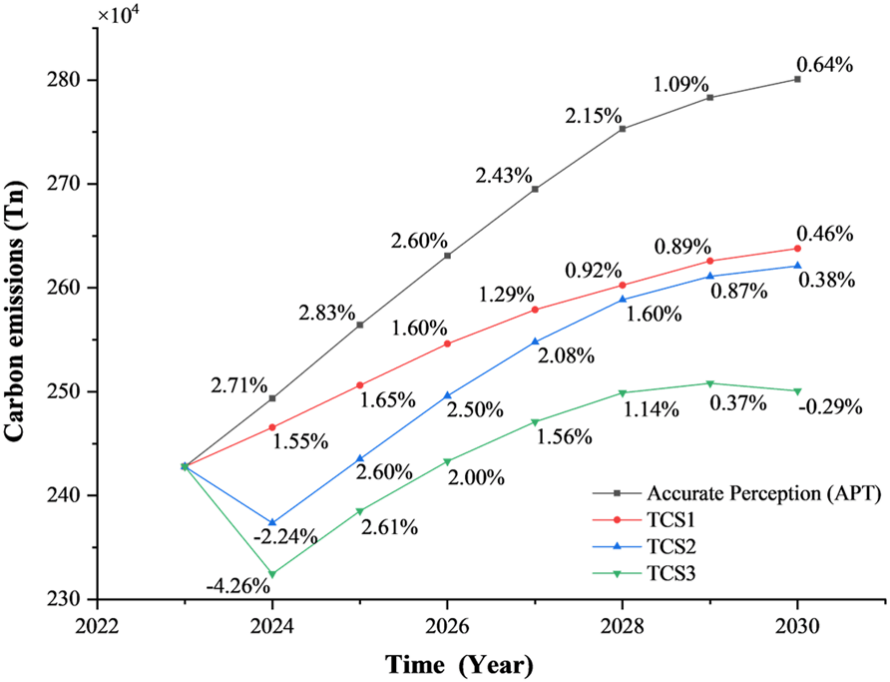
Trends in carbon emissions from urban passenger transportation under the portfolio scenario

Simulation across multiple scenarios demonstrated that no single strategy alone suffices to achieve the targeted carbon peak in passenger transportation. Therefore, integrating diverse strategies is imperative for accurately modeling the evolution of carbon emissions from passenger transportation in Lanzhou. Figure 10 presents projections combining demand management and technological interventions, illustrating total passenger transportation carbon emissions over the coming decade. Scenarios TCS1, TCS2, and TCS3 consistently yield lower emissions than the APT baseline throughout the period, with growth rates declining markedly to 0.46%, 0.38%, and –0.29%, respectively, by 2030. These outcomes indicate that the synergistic application of demand management and technological strategies can facilitate attainment of carbon peaking in passenger transportation by 2030. Detailed analysis of TCS3 reveals an emissions trajectory featuring an initial rise followed by a decline within the next ten years, peaking at 2.508 million tonnes in 2029 and subsequently decreasing to 2.501 million tonnes in 2030. Under these integrated measures, total passenger transportation carbon emissions are projected to peak in 2030, representing an annual reduction of 300 thousand tonnes relative to the APT scenario without intervention. This evidence underscores that passenger transportation policies combining travel behavior optimization, regulatory support, and technological advancement can substantially mitigate carbon emissions within the sector.

## Discussion and conclusions

### Discussion

This study developed a dynamic framework for the PCES that comprehensively assessed the impacts of passenger transport demand, management interventions, and technological strategies on urban transport carbon emissions. Nineteen operational and integrated policy combinations were proposed to support the prediction and optimization of carbon emission pathways. Results indicate that the synergy of multiple measures and policy combinations can significantly accelerate achieving the ‘carbon peak’ target in the passenger transport sector. Carbon emissions from urban passenger transport are influenced by key factors, including city population size, GDP, energy intensity, vehicle ownership, and the proportion of new energy vehicles, while also being constrained by urban form, socio-economic development level, and energy structure. Integrating travel demand, management tools, and technological measures within the PCES framework enables a thorough elucidation of the mechanisms through which these factors affect carbon emissions and the effectiveness of policy implementation, providing a foundation for formulating differentiated low-carbon transport strategies.

In contrast to previous studies that mainly focus on individual policy measures or overlook heterogeneity within urban passenger transport systems, this research introduces a dynamic PCES framework integrating DMT strategies. This framework reveals the dynamic coupling relationships between different intervention mechanisms and the evolution of their marginal effects. Prior research highlights the critical importance of promoting zero-emission vehicles and clean power systems for deep decarbonization [25], while emphasizing the need to combine technological progress with demand management strategies that account for population and regional differences [30]. Building upon these insights, this study moves beyond static assessments to capture the inherent nonlinear feedback within passenger transport systems, systematically evaluating the emission reduction effects of multi-strategy synergies. The findings show that synergistic effects of technological innovation and management pathways outperform single measures, especially in cities at intermediate development stages experiencing concurrent demand growth and energy transition,however, emission reduction benefits from technological advances may be partly offset by rigid travel demand, necessitating demand management policies. This conclusion aligns with related studies [58, 28]. Additionally, by combining the STIRPAT model with system dynamics, this framework enables multi-scenario evolution and feedback analysis, dynamically tracking system responses to policy combinations over time. This provides policymakers with a flexible and transparent analytical tool for optimizing carbon emission pathways and prioritizing resource allocation.

Although the PCES framework introduces a system dynamics model for predicting CO_2_ emissions from DMT perspectives—highlighting the influence of urban passenger transport patterns on carbon emissions and guiding China’s transportation sector toward carbon peaking—it possesses certain limitations in facilitating deeper decarbonization transformations and advancing China’s 2060 carbon neutrality goals. Firstly, the precision of carbon emissions measurement requires enhancement; future models should comprehensively incorporate factors such as fuel efficiency, road conditions, passenger turnover, unit turnover, and meteorological variables under real-world operating conditions across diverse transport modes and vehicle types to achieve more accurate emissions quantification. Secondly, the adaptability of urban transport carbon reduction strategies across varied socio-economic contexts warrants systematic validation, given that identical strategies may produce differing outcomes contingent upon economic development levels and policy environments. Consequently, emission reduction strategy combinations must be tailored and optimized according to local conditions. Moreover, this study predominantly addresses urban passenger transport, excluding freight, aviation, high-speed rail, and other long-distance transport modes. Future research should expand system boundaries to encompass regional and national transport corridors to fully capture the emission reduction potential of multi-level, multi-modal transport systems. Additionally, there is a need to deepen the characterization of resident behavioral responses and policy implementation constraints, as well as to investigate the long-term carbon impacts of emerging technologies, including autonomous driving and hydrogen-powered transportation. Through such extensions and refinements, the PCES framework could more effectively bridge theory and practice, offering increasingly adaptive and actionable decision support for low-carbon urban passenger transportation systems across different development stages.

### Conclusions

This study centered on Lanzhou, a representative inland transportation hub in China, and established a system dynamics framework to evaluate urban passenger transportation carbon emissions while validating its effectiveness. The proposed DMT framework simulated the influence mechanisms of DMT strategies on urban transportation carbon emissions, creating a comprehensive and practicable structure for forecasting emissions and optimizing development pathways in urban passenger transport. Unlike isolated strategies, this framework provides urban transportation decision-makers across diverse travel modes with a foundation to implement carbon reduction measures tailored to their cities’ unique characteristics. It also supports emission forecasting and pathway planning based on the synergistic effects of combined interventions. Findings indicate that urban passenger transportation CO_2_ emissions in Lanzhou are projected to increase, reaching 2.848 million tonnes by 2030, although the growth rate is expected to decelerate markedly after 2028. Scenario simulations of DMT measures reveal that individual strategies—such as new energy vehicle adoption and private car trip restrictions—effectively reduce emissions but only partially mitigate urban passenger transport emissions. Importantly, prioritizing public transportation significantly influences travel demand, while increasing the share of new energy vehicles offers a viable emission reduction approach. Achieving the 2030 Peak Carbon Goal necessitates a multifaceted strategy encompassing controls on fuel vehicle growth, promotion of technological innovation, and a focus on public transit prioritization to maximize impact. Accordingly, results suggest that a comprehensive combination of policies and measures can accelerate attainment of the Peak Carbon target in passenger transportation.

The potential for carbon reduction in urban passenger transportation hinges on three critical management and planning dimensions: demand, government initiatives, and technological measures. First, subsidies for new energy vehicles can stimulate market uptake and fleet upgrades aligned with rising demand. Second, ongoing development of public transportation prioritization strategies is essential to enhance convenience and safety, reducing modal conflicts and congestion. Additionally, traffic restriction policies coupled with energy technology advancements exert significant influence on public transit travel patterns and contribute to emission reductions. The integrated application of these measures can effectively steer travel behavior changes, further curbing passenger transportation carbon emissions. Nonetheless, urban transportation management and technical planning require customization based on city- and region-specific contexts. In densely populated urban areas characterized by complex travel dynamics and constrained public transit infrastructure, further validation of the effectiveness of various strategies remains necessary.

## Supplementary Information

Additional file 1.

## Data Availability

The data that support the findings of this study are available from the corresponding author upon reasonable request.
