# Supplementary material for: Assessing the effectiveness of demand-management-technology in reducing CO2 from urban passenger transportation
Source: Carbon Balance Manag. 2025 Nov 10;20:54. doi: 10.1186/s13021-025-00343-y (PMC12604176; doi:10.1186/s13021-025-00343-y)
Supplement: Supplementary file 1 — Additional file 1. [file 13021_2025_343_MOESM1_ESM.docx]

Supplementary Materials for

**Assessing the effectiveness of demand-management-technology in reducing CO_2_ from urban passenger transportation**

Xin Li^a^, Yongsheng Qian^a,∗^, Jianxin Wang^a^, Minan Yang^a,b^, Junwei Zeng^a^ , Xiaofang Xie^c^

*a School of Traffic and Transportation, Lanzhou Jiaotong University, Lanzhou, 730070, China;*

*b School of Architecture and Urban Planning, Lanzhou Jiaotong University, Lanzhou, 730070, China;*

*c School of Tourism and History and Culture, YiLi Normal University, Xinjiang -Yining, 835000, China;*

∗Corresponding author at: No. 88, Anning West Road, Anning District, Lanzhou City, Gansu Province, China

*Email addresses:* [13230003@stu.lzitu.edu.cn(X.Li.)](mailto:13230003@stu.lzitu.edu.cn(X.Li.)); [qianyongsheng@mail.lzjtu.cn(Y.Qian.)](mailto:qianyongsheng@mail.lzjtu.cn(Y.Qian.)); [wxx203301510@163.com(J.Wang.)](mailto:wxx203301510@163.com(J.Wang.)); [yangminan@mail.lzjtu.cn(M.Yang.)](mailto:yangminan@mail.lzjtu.cn(M.Yang.)); [zengjunwei@mail.lzjtu.cn(J.Zeng.)](mailto:zengjunwei@mail.lzjtu.cn(J.Zeng.)); 573973094[@qq.com(X.Xie.)](mailto:zengjunwei@mail.lzjtu.cn(J.Zeng.)).

1 Materials and Methods

This paper employs a system dynamics approach to construct a system dynamics model of carbon emissions from urban passenger transport. The model comprises three core components: travel demand, management policies, and technological innovation (DMT). Through state variables, rate variables, and auxiliary variables, the model characterises the dynamic coupling relationships among economic development, travel behaviour, energy consumption, and carbon emissions within the transport system. The aim is to simulate the evolution paths and response mechanisms of carbon emissions under multiple policy scenarios. The simulation period is set from 2011 to 2022, with a time step of $DT=1$ year. The symbols for model variables are defined as follows: state variables are denoted by $L$, rate variables by $T$, and auxiliary variables by $A$. The time suffixes $J$, $K$, and $L$ correspond to past, present, and future time points, respectively. $LM$ represents the state equation, $RM$ the rate equation, and $AM$ the auxiliary equation.

**1.1 Travel demand**

Residents' travel demand is primarily influenced by the combined effects of socio-economic development levels and transportation supply conditions, particularly driven by macroeconomic factors such as economic growth, population size, and infrastructure development levels. Urban passenger transport carbon emissions are influenced by passenger transport turnover and the carbon emission intensity per unit of turnover. In the model, travel demand is characterised through two subsystems: the socio-economic subsystem and the passenger transport subsystem. The core objective is to quantify how changes in demand influence carbon emissions through transportation supply and infrastructure levels, and to provide foundational parameter support for ICL and PTP strategies in scenario simulations.

(1) Socio-economic subsystem.

The socio-economic subsystem is the basic dynamic module of the passenger transport carbon emission system dynamics model and is the core driving force behind the growth in transport demand. Economic development is closely related to population size, and per capita GDP is considered an important economic indicator determining changes in passenger transport turnover. Additionally, to support passenger transport development, local governments typically increase infrastructure investment to improve transport conditions from the supply side and promote low-carbon transformation and carbon reduction through specialised funding. This subsystem is primarily used to analyse the dynamic relationship between regional GDP and infrastructure development levels (as shown in Table S1).

**Table S1:** Main variables of the economic subsystem

| System Variables | Variable Name | Type | Unit |
| --- | --- | --- | --- |
| GDPT | Gross Domestic Product (Regional) | State Variable | 10,000 yuan |
| GDPG | GDP Growth | Rate Variable | 10,000 yuan |
| ICI | Infrastructure Construction Investment | Auxiliary Variable | 10,000 yuan |
| ICR | Infrastructure Investment Coefficient | Auxiliary Variable | Dmnl |
| PTCI | Passenger Transport Carbon-Reduction Investment | Auxiliary Variable | 10,000 yuan |
| PTCR | Carbon-Reduction Investment Coefficient | Auxiliary Variable | Dmnl |
| TSVA | Tertiary Industry Added Value | Table Function | 10,000 yuan |
| GDPR | GDP Growth Rate | Table Function | Dmnl |
| PTVA | Passenger Transport Added Value | Auxiliary Variable | 10,000 yuan |
| CTA | Carbon Tax Amount | Auxiliary Variable | 10,000 yuan |
| ICC | Infrastructure Level | Auxiliary Variable | Dmnl |
| CST | Number of Charging Piles | Auxiliary Variable | Unit (pcs) |
| RMT | Road Mileage | Auxiliary Variable | km |
| RTT | Rail Transit Mileage | Auxiliary Variable | km |
| BNT | Bus Network Length | Auxiliary Variable | km |
| Time | Time | Shadow Variable | year |

The main equation is as follows:

$${LM}_{1}\text{: }GDPT.K=GDPT.J+GDPG\times DT$$

$${RM}_{1}\text{: }GDPG=GDPT\times GDPR+PTVA+STEP\left( CTA,2020 \right)$$

$${AM}_{1}\text{: }PTCI=GDPT\times PTCR$$

$${AM}_{2}\text{: }ICI=GDPT\times ICR$$

$${AM}_{3}\text{: }PTVA=f\left( TSVA,CTA,ECT \right)$$

$${AM}_{4}: ICC=f\left( RMT,RTT,BNT,CST \right)$$

${LM}_{1}$ represents the dynamic changes in regional GDP as it accumulates over time; ${RM}_{1}$ indicates that GDP growth is jointly influenced by GDP growth rate, passenger transport value added, and carbon tax policy; ${AM}_{1-2}$ is used to describe the functional relationship between infrastructure investment, transport carbon reduction investment, and GDP.

where, ${AM}_{3}$ introduces the Cobb–Douglas function to analyse the elastic effects of tertiary industry value-added, carbon taxes, and energy consumption on passenger transport value-added. $\delta_{i}^{n}$ respectively represent the elasticity coefficients of carbon tax structure on passenger transport energy consumption and passenger transport value-added, $\varepsilon_{1}$ denotes the carbon tax ratio, and $\varepsilon_{2}$ denotes the ratio of other taxes.

$$InPTVA=\sum\delta_{i}^{1}\varepsilon_{1}InTSVA+\sum\delta_{i}^{2}\varepsilon_{2}CTA+\sum\delta_{i}^{3}\varepsilon_{2}ECT$$

To further characterise the relationship between infrastructure level (ICC) and passenger transport network characteristics, a linear regression analysis was conducted using road mileage, bus network length, rail transit mileage, and charging station number as independent variables. The results are shown in Table S2, with R² = 0.941 and p ≤ 0.05 for each independent variable being statistically significant.

**Table S2:** Regression analysis of basic design levels

| Variables | Non-standardised coefficient | P | R^2^ |
| --- | --- | --- | --- |
| Road mileage | 0.916 | 0.033 |  |
| Public transport network length | 0.533 | 0.001 |  |
| Rail transit mileage | 0.318 | 0.019 |  |
| Number of charging stations | 0.104 | 0.027 |  |
| Infrastructure investment | 0.412 | 0.025 |  |
| Constants | 1583.5 | 0.038 | 0.951 |

(2) Urban Passenger Transport Subsystem.

The urban passenger transport subsystem is used to characterise the direct impact of passenger transport turnover and public transport priority policies on carbon emissions. Through interactions with the socio-economic subsystem and the energy consumption subsystem, it reveals the transmission mechanisms through which changes in transport supply and demand affect carbon emissions. This subsystem, on the one hand, reflects the dynamic balance between passenger demand and supply, using passenger transport turnover as the core indicator to comprehensively analyse changes in passenger flow across various transport modes; on the other hand, it depicts the feedback effects of traffic management and congestion levels on public transport efficiency, with a focus on evaluating the dynamic roles of ‘public transport priority policies’ (PTP scenario) and traffic management measures (RPR scenario) in alleviating congestion and improving transport efficiency. Specific information is shown in Table S3.

**Table S3:** Main variables of the urban passenger transport subsystem

| System Variables | Variable Name | Type | Unit |
| --- | --- | --- | --- |
| PT | Passenger Turnover | State Variable | 10,000 person-kilometers |
| PTVT | Total Public Transport Passenger Volume | State Variable | 10,000 persons |
| PTVG | Increase in Public Passenger Volume | Rate Variable | 10,000 persons |
| PTVD | Decrease in Public Passenger Volume | Rate Variable | 10,000 persons |
| PD | Passenger Demand | Auxiliary Variable | 10,000 persons |
| PTR | Transport Efficiency | Auxiliary Variable | Dmnl |
| CCR | Conversion Coefficient | Auxiliary Variable | Dmnl |
| TCS | Traffic Congestion Level | Auxiliary Variable | Dmnl |
| TCSD | Traffic Congestion Delay Time | Auxiliary Variable | days |
| PTP | Public Transport Priority Policy (PTP) | Auxiliary Variable | Dmnl |
| TC | Traffic Management | Auxiliary Variable | Dmnl |
| TCD | Traffic Management Delay Time | Auxiliary Variable | Dmnl |
| Time | Time | Shadow Variable | year |

The main equation is as follows:

$${LM}_{2}\text{: }PTVT.K=PTVT.J+(PTVG.JK-PTVD.JK)\times DT$$

$${RM}_{2}\text{: }\text{PTVG}=\mu_{1}\text{TAN}+\mu_{2}\text{RTN}+\mu_{3}\text{BUN}\times\text{PTP}+\mu_{4}\times\text{DELAY3}(\text{TCS,TCSD})$$

$${RM}_{3}\text{: }PTVD=\mu_{5}\times PCN$$

$${AM}_{5}\text{: }PT.K=PD-IFTHENELSE(PD<1,ICC\times PTR,0)$$

$${AM}_{6}\text{: }PD=f(TAVA)$$

$${AM}_{7}\text{: }\text{PTR=ICC/PD}$$

$${AM}_{8}\text{: }\text{TCS=}\mu_{7}\text{PT+}\mu_{8}\text{EBN+}\mu_{9}\text{DELAY1}(\text{TC},\text{TCD})+\mu_{\text{10}}\text{FTN}$$

${LM}_{2}$ is used to describe the dynamic characteristics of the total public transport passenger volume over time, with changes influenced by the difference between the increase and decrease in public transport passenger volume. ${RM}_{2}$ indicates that the increase in public transport passenger volume is jointly influenced by factors such as the number of buses, rail transit vehicles, and taxis, as well as traffic congestion levels. The introduction of the third-order delay function DELAY3 simulates the lag effect of congestion. ${RM}_{3}$ is used to reflect that the decrease in public passenger transport is primarily influenced by changes in the total number of private vehicles, where the parameter μ₅ represents the substitution effect of private vehicle diversion on public transport. $AM$ is used to describe the interactive relationship between passenger demand and supply efficiency, as well as the feedback mechanism between transportation efficiency and congestion levels, and combines the ‘public transport priority policy’ (PTP) and ‘traffic management strategy’ (RPR) to dynamically adjust the traffic flow structure of passenger travel.

To obtain the parameter value $u_{1}$ between variables, an elasticity coefficient model was constructed based on the relationship between traffic congestion levels, total public transport passenger volume, and total private car volume. The three-stage least squares method was used to obtain the elasticity coefficients between variables. The dynamic relationship formula of the model is as follows:

$$(PTVT)_{t}=\theta^{m}(PTVT)_{t-1}+\theta^{mp}(TCS)+\omega_{t}^{m}$$

$$(TCS)_{t}=\theta^{n}(TCS)_{t-1}+\theta^{nm}+\omega_{t}^{n}$$

$$\omega_{t}^{k}=\varphi^{k}\omega_{t-1}^{k}+\varepsilon_{t}^{k},k=m,n,p$$

**1.2 Policy Support**

(1) Residential Travel Subsystem.

The Residential Travel Subsystem is primarily used to describe changes in travel patterns between private vehicles and public transportation, as well as the response mechanisms to policy constraints. It focuses on analysing the distribution characteristics of residential travel demand across various transportation modes (public transportation, taxis, private vehicles) and the dynamic impacts of policy interventions (traffic restrictions, number plate restrictions, fuel price adjustments, and subsidies for new energy vehicles) on the evolution of transportation structures. The variable information for this subsystem is shown in Table S4.

**Table S4:** Main variables of the resident travel demand subsystem

| System Variable | Variable Name (English) | Unit | System Variable | Variable Name (English) | Unit |
| --- | --- | --- | --- | --- | --- |
| PCN | Total Private Cars | 10,000 vehicles | PTVTD | Public Transport Passenger Volume Delay Time | years |
| PCTD | Private Car Travel Distance | 10,000 km | ERP | New Energy Vehicle Replacement Policy | Dmnl |
| BUN | Total Buses | 10,000 vehicles | FCI | Increment of Fuel Private Cars | 10,000 vehicles |
| TAN | Total Taxis | 10,000 vehicles | ECI | Increment of Pure Electric Private Cars | 10,000 vehicles |
| PCNG | Increase in Private Cars | 10,000 vehicles | FCN | Number of Fuel Private Cars | 10,000 vehicles |
| PCND | Decrease in Private Cars | 10,000 vehicles | ECN | Number of Pure Electric Private Cars | 10,000 vehicles |
| TDG | Increase in Travel Distance | 10,000 km | PRP | Purchase Restriction Policy | Dmnl |
| TDD | Decrease in Travel Distance | 10,000 km | NEPS | New Energy Vehicle Purchase Subsidy | Dmnl |
| BUNG | Increase in Buses | 10,000 vehicles | CFD | Charging Facility Improvement Level | Dmnl |
| BUND | Decrease in Buses | 10,000 vehicles | FC | Fuel Cost | yuan/L |
| TANG | Increase in Taxis | 10,000 vehicles | FCD | Fuel Cost Delay Time | years |
| TAND | Decrease in Taxis | 10,000 vehicles | FTI | Increment of Fuel Taxis | 10,000 vehicles |
| RTM | Rail Transit Travel Distance | 10,000 km | GTI | Increment of Gas Taxis | 10,000 vehicles |
| TAM | Taxi Travel Distance | 10,000 km | ETI | Increment of Electric Taxis | 10,000 vehicles |
| BUM | Bus Travel Distance | 10,000 km | TASF | Taxi Scrapping Coefficient | Dmnl |
| TASP | Scrapping Policy | Dmnl | FCD | Fuzzy Cost Difference | Dmnl |
| FTN | Number of Fuel Taxis | 10,000 vehicles | FIC | Fuzzy Infrastructure Convenience | Dmnl |
| RTN | Total Rail Transit | 10,000 vehicles | CTCD | Cost Difference Between Private and Public Transport | Dmnl |
| FBI | Increment of Fuel Buses | 10,000 vehicles | PCTC | Private Car Travel Cost | yuan/km |
| GBI | Increment of Gas Buses | 10,000 vehicles | PTAC | Average Public Transport Travel Cost | yuan/km |
| EBI | Increment of Electric Buses | 10,000 vehicles | TATC | Taxi Travel Cost | yuan/km |
| EBN | Number of Electric Buses | 10,000 vehicles | RTC | Metro Travel Cost | yuan/km |
| NEBU | Public Transport New Energy Policy | Dmnl | BUTC | Bus Travel Cost | yuan/km |
| RMTD | Road Mileage Delay Time | years | TD | Travel Distance | 10,000 km |
| PCSR | Private Car Travel Share Rate | Dmnl | TS | Travel Satisfaction | Dmnl |
| DU | Demand Utility | Dmnl | PSN | Number of Parking Spaces | 10,000 spaces |
| SU | Supply Utility | Dmnl | Time | Time | years |

The main equation is as follows:

$${LM}_{3}\text{: }\text{PCN}.\text{K=PCN}.\text{J+}\left( \text{PCNG}.\text{JK-PCND}.\text{JK} \right)\times\text{DT}$$

$${LM}_{4}\text{: }\text{TAN}.\text{K=TAN}.\text{J+}\left( \text{TANG}.\text{JK-TAND}.\text{JK} \right)\times\text{DT}$$

$${LM}_{5}\text{: }\text{BUN}.\text{K=BUN}.\text{J+}\left( \text{BUNG}.\text{JK-BUND}.\text{JK} \right)\times\text{DT}$$

$${LM}_{6}\text{: }\text{PCTD}.\text{K=PCTD}.\text{J+}\left( \text{TDG}.\text{JK-TDD}.\text{JK} \right)\times\text{DT}$$

$${RM}_{4}: PCNG\text{=FCI+ECI}$$

$${RM}_{5}\text{: }\text{PCND}=\rho_{1}\text{BUN}+\rho_{2}\text{DELAY1}\left( \text{FC,FCD} \right)$$

$${RM}_{6}\text{: }\text{TANG=FTI+GTI+ETI}$$

$${RM}_{7}\text{: }TAND=TAN\times TASF$$

$${RM}_{8}\text{: }\text{BUNG=FBI+GBI+EBI}$$

$${RM}_{9}\text{: }BUND=\rho_{3}RTN$$

$${RM}_{10}\text{: }\text{TDG}=\rho_{4}\text{GDPT}+\rho_{5}\text{PCN}+\rho_{6}\text{PCSR}+\rho_{7}\text{DELAY1}\left( \text{RMT},\text{RMTD} \right)$$

$${RM}_{11}\text{: }\text{TDD}=\rho_{8}\text{BUM}+\rho_{9}\text{TAM}+\rho_{\text{10}}\text{BUM}{+\rho}_{\text{11}}\text{DELAY}\text{1}\left( \text{FC},\text{FCD} \right)+\rho_{12}\text{DELAY1}\left( \text{PATVT,PATVTD} \right)$$

$${AM}_{10}\text{: }FCI=FCN\times PRP$$

$${AM}_{11}\text{: }\text{ECI=ECN}\times\text{NEPS}\times\text{CFD}$$

$${AM}_{12}\text{: }FTI=FTN\times ERP$$

$${AM}_{13}\text{: }EBI=EBN\times NEBU$$

$${AM}_{14}\text{: }\text{PCSR=f}\left( \text{DU,SU} \right)$$

$${RM}_{15}\text{: }\text{T}\text{S}=\rho_{13}\text{PSN}+\rho_{14}\text{TCS}$$

$${AM}_{16}\text{: }\text{PTAC=}\left( \text{TATC+RTC+BUTC} \right)\text{/3}$$

$${AM}_{17}\text{: }\text{PCTC=}\text{f}\text{1}\left( \text{TD} \right)$$

$${AM}_{18}\text{: }\text{TATC=f2}\left( \text{TD} \right)$$

$${AM}_{19}\text{: }\text{RTC=f3}\left( \text{TD} \right)$$

$${AM}_{20}\text{: }\text{BUTC=f4}(\text{TD})$$

${LM}_{3}$ represents the dynamic changes in the total number of private vehicles, driven by both increases and decreases in the number of private vehicles; ${LM}_{4}$ and ${LM}_{5}$ describe the changes in the total number of taxis and buses, respectively; ${LM}_{6}$ is used to depict the evolution of private vehicle travel distances over time. ${RM}_{4}$, ${RM}_{6}$, and ${RM}_{8}$ calculate the increase in private cars, taxis, and buses, respectively, primarily influenced by the number of fuel-powered vehicles and new energy vehicles, as well as related policies (such as new energy vehicle replacement policies and public transport new energy policies); ${RM}_{5}$, ${RM}_{7}$, and ${RM}_{9}$ describe the corresponding decreases in travel distances for each mode of transport, considering the effects of factors such as scrappage policies, fuel prices, and rail transport supply; ${RM}_{10}$, ${RM}_{11}$ characterises changes in travel distance, driven by multiple factors such as GDP, private car ownership, road mileage, and fuel prices, and reflects the lag in policy effects through a first-order lag function. ${AM}_{10-13}$ describe the incremental calculations for different types of vehicles, such as the relationship between the increase in fuel-powered private cars and the purchase restriction policy, and the relationship between the increase in pure electric private cars and new energy vehicle subsidies and charging infrastructure; ${AM}_{14}$ depicts the feedback relationship between private vehicle travel share and demand utility and supply utility; ${AM}_{16-20}$ Provides parameters for travel decision-making and cost difference analysis by comprehensively calculating the travel costs of different modes of transportation.

**1.3 Technological Innovation**

(1) Energy Consumption and Carbon Emissions Subsystem.

The Energy Consumption and Carbon Emissions Subsystem is the model's results and constraints module, with its core focus on capturing the regulatory effects of technological progress on energy consumption and carbon emissions intensity. This subsystem models the energy consumption levels and carbon emission coefficients of vehicles using different energy types, such as gasoline, diesel, natural gas, and electric vehicles, to assess the long-term impact of improvements in energy efficiency, optimisation of energy structure, and advancements in carbon reduction technologies on carbon emissions. It primarily analyses the dynamic changes in total energy consumption and total carbon dioxide emissions, with specific variables as shown in Table S5.

**Table 5:** Main variables of the energy consumption and carbon emissions subsystem

| System Variable | Variable Name (English) | Type | Unit |
| --- | --- | --- | --- |
| ECT | Total Energy Consumption | Stock Variable | 10,000 tons of coal eq. |
| CDET | Total Carbon Dioxide Emissions | Stock Variable | 10,000 tons |
| ECG | Energy Consumption Increase | Rate Variable | 10,000 tons of coal eq. |
| ECD | Energy Consumption Decrease | Rate Variable | 10,000 tons of coal eq. |
| CDEG | Carbon Emission Increase | Rate Variable | 10,000 tons |
| CDED | Carbon Emission Decrease | Rate Variable | 10,000 tons |
| GCEC | Gasoline Vehicle Energy Consumption | Auxiliary Var. | t/10⁴ t·km |
| DCEC | Diesel Vehicle Energy Consumption | Auxiliary Var. | t/10⁴ t·km |
| NGEC | Natural Gas Vehicle Energy Consumption | Auxiliary Var. | t/10⁴ t·km |
| ECEC | Electric Vehicle Energy Consumption | Auxiliary Var. | kWh |
| GTN | Number of Gas Taxis | Auxiliary Var. | vehicles |
| GBN | Number of Gas Buses | Auxiliary Var. | vehicles |
| ETN | Number of Pure Electric Taxis | Auxiliary Var. | vehicles |
| FBN | Number of Fuel Buses | Auxiliary Var. | vehicles |
| RTEC | Rail Transit Power Consumption | Auxiliary Var. | kWh |
| GCEF | Gasoline Vehicle Emission Factor | Auxiliary Var. | Dmnl |
| DCEF | Diesel Vehicle Emission Factor | Auxiliary Var. | Dmnl |
| NGEF | Natural Gas Vehicle Emission Factor | Auxiliary Var. | Dmnl |
| ECEF | Electric Vehicle Emission Factor | Auxiliary Var. | Dmnl |
| CEDR | Carbon Reduction Coefficient | Auxiliary Var. | Dmnl |
| CTR | Carbon Tax Rate | Auxiliary Var. | Dmnl |
| EEF | Energy Efficiency | Auxiliary Var. | Dmnl |

The main equation is as follows:

$${LM}_{7}\text{: }\text{ECT}.\text{K=ECT}.\text{J+}\left( \text{ECG}.\text{JK-ECD}.\text{JK} \right)\times\text{DT}$$

$${LM}_{8}\text{: }CDET.K=CDET.J+\left( CDEG.JK-CDED.JK \right)\times DT$$

$${RM}_{12}\text{: }\text{ECG}=\mu_{1}\text{GCEC}+\mu_{2}\text{DCEC}+\mu_{3}\text{NGEC}+\mu_{4}\text{ECEC}+\mu_{5}\text{HEVEC}$$

$${RM}_{13}\text{: }ECD=ECT\times EEF$$

$${RM}_{14}\text{: }\text{CDEG}=\text{GCEC}\times\text{GCEF}+\text{DCEC}\times\text{DCEF}+\text{NGEC}\times\text{NGEF}+\text{ECEC}\times\text{ECEF}$$

$${RM}_{15}\text{: }CDED=DELAY1\left( CEDR,2 \right)\times PTCI$$

$${AM}_{21}\text{: }EEF=DELAY3\left( \left( \frac{CTA}{3.161\times ECT\times13.89}\times\vartheta\left( CTR \right) \right),1 \right)$$

$${AM}_{22}\text{: }\text{GCEC}=\varphi_{1}\omega_{1}\text{FCN}+\varphi_{2}\omega_{2}\text{FTN}$$

$${AM}_{23}\text{: }NGEC=\varphi_{3}\omega_{2}GTN+\varphi_{4}\omega_{3}FBN$$

$${AM}_{24}\text{: }\text{ECEC}=\varphi_{5}\omega_{1}\text{ECN}+\varphi_{6}\omega_{2}\text{ETN}+\varphi_{7}\omega_{3}\text{EBN}+\text{RTEC}$$

$${AM}_{25}\text{: }DCEC=\varphi_{8}\omega_{3}FBN$$

${LM}_{7}$ represents the total energy consumption over time, equal to the total energy consumption at the previous moment plus the difference between the increase and decrease in energy consumption; ${LM}_{8}$ describes the dynamic changes in total carbon dioxide emissions, influenced by the increase and decrease in carbon emissions. ${RM}_{12}$ calculates the increase in energy consumption, composed of the energy consumption of gasoline, diesel, natural gas, and electric vehicles, along with their conversion coefficients; ${RM}_{13}$ reflects the amount of energy saved, determined by the total energy consumption and energy efficiency; ${RM}_{14}$ calculates the increase in carbon emissions by weighting and summing the energy consumption of different energy types with their corresponding emission coefficients; ${RM}_{15}$ describes the relationship between the reduction in carbon emissions and carbon reduction investments, and reflects the lag in investment effects through the DELAY1 function. ${AM}_{21}$ describes the relationship between energy-saving efficiency, energy consumption, carbon tax amounts, and carbon tax rates, and introduces the lagged characteristics of energy-saving technology effects through the DELAY3 function; ${AM}_{22-25}$ respectively depict the energy consumption calculation methods for different types of vehicles. This subsystem is directly related to the technological innovation scenarios (ENI, EMI).
